# Microplastics and Nanoplastics in the Human Diet: Sources of Exposure, Bioavailability, Toxicokinetics, and Systemic Health Effects

**DOI:** 10.3390/molecules31172945

**Published:** 2026-08-22

**Authors:** Łukasz Kogut, Czesław Puchalski, Julia Jastrzębska, Grzegorz Zaguła

**Affiliations:** 1 Department of Bioenergetics, Food Analysis and Microbiology, Institute of Food Technology and Nutrition, Faculty of Technology and Life Science, University of Rzeszów, Aleja Rejtana 16C, 35-959 Rzeszów, Poland; lukasz.kogut2@wp.pl (Ł.K.); cpuchal@ur.edu.pl (C.P.); 2 Medical Centre in Łańcut LLC, Ignacego Paderewskiego 5, 37-100 Łańcut, Poland; j.jastrzebska2105@gmail.com

**Keywords:** microplastics, nanoplastics, dietary exposure, food contamination, bioavailability, toxicokinetics, bioaccumulation, systemic toxicity, human health, oxidative stress, inflammation

## Abstract

**Background/Objectives**: Microplastics (MPs) and nanoplastics (NPs) have emerged as ubiquitous environmental contaminants resulting from the extensive production, use, and degradation of plastic materials. Human exposure occurs primarily through contaminated food and drinking water, with inhalation representing an additional important route. Growing concern has focused on the ability of these particles, particularly NPs, to cross biological barriers, enter the systemic circulation, and reach human tissues. The aim of this review was to summarize current evidence on dietary exposure to MPs and NPs, their gastrointestinal bioavailability and toxicokinetics, and their potential systemic health effects, with particular emphasis on organ-specific responses, underlying biological mechanisms, and the strength and limitations of the available evidence. **Methods**: A comprehensive narrative review of the scientific literature published between 2000 and 2026 was conducted using PubMed/MEDLINE, Scopus, Web of Science, and Google Scholar. Original research articles and review papers addressing dietary exposure, occurrence in food and drinking water, migration from food-contact materials, gastrointestinal absorption, translocation, biodistribution, bioaccumulation, elimination, molecular mechanisms, and potential organ-specific or systemic health effects were included. Publications without full-text availability, conference proceedings, editorials, commentaries, duplicate publications, and studies without relevance to human exposure or health were excluded. **Results**: Food, drinking water, beverages, and food-contact materials represent important sources of human exposure to MPs and NPs. Following ingestion, most larger particles are eliminated through the gastrointestinal tract, whereas smaller MPs and particularly NPs may cross biological barriers and potentially reach the systemic circulation and distant tissues. Experimental studies consistently identify interconnected biological responses involving oxidative stress, inflammation, mitochondrial dysfunction, barrier impairment, immune dysregulation, genotoxicity, apoptosis, and endocrine disruption. These mechanisms have been associated with alterations in the gastrointestinal, respiratory, cardiovascular, nervous, urinary, reproductive, endocrine, and skeletal systems and with biological processes relevant to carcinogenesis. However, most mechanistic evidence derives from in vitro and animal models, whereas human evidence remains limited and predominantly observational. Consequently, the extent to which these experimental findings translate into clinically significant effects in humans remains uncertain. **Conclusions**: Current evidence supports the biological plausibility of systemic effects associated with MNP exposure but is insufficient to establish causal relationships between chronic dietary exposure and specific human diseases. The detection of MNPs in human tissues and reported associations with pathological conditions should therefore be interpreted cautiously. Standardized analytical methods, improved characterization of realistic human exposure, and well-designed longitudinal epidemiological studies integrating quantitative exposure assessment with validated clinical outcomes are required to clarify dose–response relationships, long-term health effects, and the clinical significance of MNP exposure.

## 1. Introduction

Plastics are currently among the most important materials used by modern society [1,2,3,4]. Their widespread use results from their advantageous physicochemical properties, including low weight, high durability, corrosion resistance, and low production costs [1,5,6]. However, the rapid expansion of the plastics industry since the mid-twentieth century has led to a dramatic increase in the accumulation of plastic waste in the environment [1,4,7,8]. Due to their limited biodegradability, most plastics persist in ecosystems for decades or even centuries, gradually fragmenting into increasingly smaller particles [3,9].

This process has resulted in the formation of micro- and nanoplastics, which are now recognized as some of the most widespread environmental contaminants [10,11,12,13]. These particles have been detected in surface waters, groundwater, soils, the atmosphere, food products, and human and animal tissues [2,14,15]. A growing body of evidence indicates that human exposure to micro- and nanoplastics is continuous and affects virtually all population groups [4,16,17,18,19]. Of particular concern is their potential to penetrate biological tissues and affect cellular and organ function [10,17]. Consequently, micro- and nanoplastics have become the subject of intensive research in toxicology, public health, and environmental medicine [4,10,20].

### 1.1. Characteristics of Micro- and Nanoplastics

Micro- and nanoplastics (MNPs) are small plastic particles originating either from the degradation of larger plastic debris or from intentional production for specific industrial and consumer applications [3,6,21,22]. Microplastics are generally defined as plastic particles smaller than 5 mm, whereas nanoplastics comprise particles with dimensions below 1 µm, although more restrictive definitions commonly classify NPs within the 1–100 nm range [9,22,23]. Thus, the 5 mm threshold represents the conventionally accepted upper size limit for microplastics. The lack of a universally accepted definition of nanoplastics primarily reflects the limitations of current analytical methods and the continuing development of techniques capable of detecting and characterizing the smallest particles [5,20,24].

Based on their origin, microplastics are classified as primary microplastics, which are intentionally manufactured at microscopic dimensions, and secondary microplastics, which are generated through the degradation of larger plastic materials under the influence of ultraviolet radiation, oxidative processes, mechanical abrasion, and microbial activity [10,11,23]. Secondary microplastics are currently considered the predominant fraction of microplastics present in the environment [4,11,25]. The most frequently identified polymers include polyethylene (PE), polypropylene (PP), polystyrene (PS), polyethylene terephthalate (PET), and polyvinyl chloride (PVC) [5,6,26]. In addition to the polymer matrix itself, micro- and nanoplastics may contain numerous technological additives, including plasticizers, stabilizers, flame retardants, and pigments, which may gradually leach into the environment and potentially contribute to human exposure [1,2,18].

A characteristic feature of MNPs is their hydrophobic surface and high surface-area-to-volume ratio, particularly in the case of nanoplastics [1,2,23]. These properties facilitate the adsorption of heavy metals, pesticides, persistent organic pollutants, and microorganisms. Consequently, micro- and nanoplastics may act as carriers of potentially toxic substances and facilitate their transport into biological systems, a phenomenon commonly referred to as the “Trojan horse” effect [3,6,21,27].

### 1.2. Sources and Routes of Human Exposure

Human exposure to micro- and nanoplastics is widespread and affects virtually all population groups [3,10,16]. These particles have been detected in food, drinking water, atmospheric air, and numerous everyday consumer products, making complete avoidance of exposure virtually impossible under current environmental conditions [6,10]. The oral route is considered one of the principal pathways of human exposure, as micro- and nanoplastics have been identified in fish, seafood, table salt, honey, milk, beverages, and drinking water from both municipal supplies and bottled sources [2,3,21,28]. An additional source of dietary exposure is the release and migration of plastic particles from food-contact materials during food storage and thermal processing [4,10,29,30,31,32,33]. The major dietary sources and pathways of human exposure to MPs and NPs are summarized in Table 1.

The inhalation route also represents a relevant pathway of human exposure [15,16,20]. Microplastics are present in both outdoor and indoor environments, with airborne plastic particles originating primarily from synthetic textile fibers, tire wear, building materials, and industrial activities [4,10,16,30]. The smallest particles may reach the pulmonary alveoli and potentially enter the systemic circulation [2,28]. Dermal exposure is generally considered less significant; however, nanoplastics may penetrate damaged epidermis, hair follicles, and sweat gland ducts, particularly following contact with cosmetic products or clothing made from synthetic fibers [2,14,18].

### 1.3. Toxicokinetics of Micro- and Nanoplastics

The toxicokinetics of micro- and nanoplastics encompasses the processes of absorption, distribution, bioaccumulation, and elimination from the human body [13,27]. Absorption depends primarily on particle size, shape, chemical composition, and surface characteristics [3,10,19,21,25]. Polymer composition may additionally influence MNP toxicokinetics by determining physicochemical properties such as hydrophobicity, density, surface chemistry, and interactions with biological molecules. These properties can modify particle dispersion, adsorption of biomolecules and contaminants, membrane interactions, cellular uptake, and subsequent tissue distribution. Surface properties appear particularly relevant for NPs, as surface charge and functionalization can influence interactions with biological membranes, biomolecular corona formation, cellular internalization, and subsequent biological responses. Nevertheless, direct comparative evidence demonstrating polymer-specific differences in absorption, biodistribution, and elimination under equivalent experimental conditions remains limited, as most studies investigate individual polymer types rather than systematically comparing different polymers [3,19,21,25]. Within the gastrointestinal tract, the smallest particles exhibit the greatest potential for translocation and may be transported through microfold (M) cells located in Peyer’s patches or cross the intestinal epithelium via enterocytes [5,18]. In the respiratory system, nanoplastics may reach the pulmonary alveoli and cross the alveolar–capillary barrier, whereas dermal penetration occurs predominantly at sites where the epidermal barrier has been compromised [18,28,31].

Following entry into the systemic circulation, MNPs may be transported via the blood and lymphatic systems to multiple organs [3,32]. Their presence has been reported in human blood, lungs, liver, spleen, kidneys, heart, placenta, and brain, while experimental evidence indicates that nanoplastics may cross both the blood–brain and placental barriers. Evidence for MNP occurrence in internal organs is derived from both analyses of human biological samples and experimental studies. In humans, MNPs have been directly detected in collected blood and tissue samples, including lung, liver, kidney, placental, and brain tissues. In contrast, controlled in vivo animal studies have been used primarily to investigate particle translocation, biodistribution, and the ability of smaller particles, particularly NPs, to cross biological barriers. Therefore, the detection of MNPs in human tissues provides evidence of their presence but does not by itself establish the exposure route, absorbed dose, or kinetics of tissue accumulation [4,14,18,20]. Their potential for bioaccumulation is associated with high chemical stability and resistance to enzymatic degradation, which may facilitate the persistence of particles within biological tissues [3,21,23]. Micro- and nanoplastics have also been detected in human breast milk and neonatal meconium, suggesting that exposure may begin as early as the prenatal and perinatal periods [5,20,27].

Elimination occurs predominantly through the gastrointestinal tract, as the majority of ingested particles are excreted in feces [2,28,33]. Smaller particles may potentially be eliminated via the kidneys in urine, whereas particles deposited in the respiratory tract are removed through mucociliary clearance and the phagocytic activity of alveolar macrophages [5,21,23,27]. The principal processes determining the absorption, systemic distribution, tissue accumulation, and elimination of MPs and NPs are summarized in Table 2.

**Figure 1:**
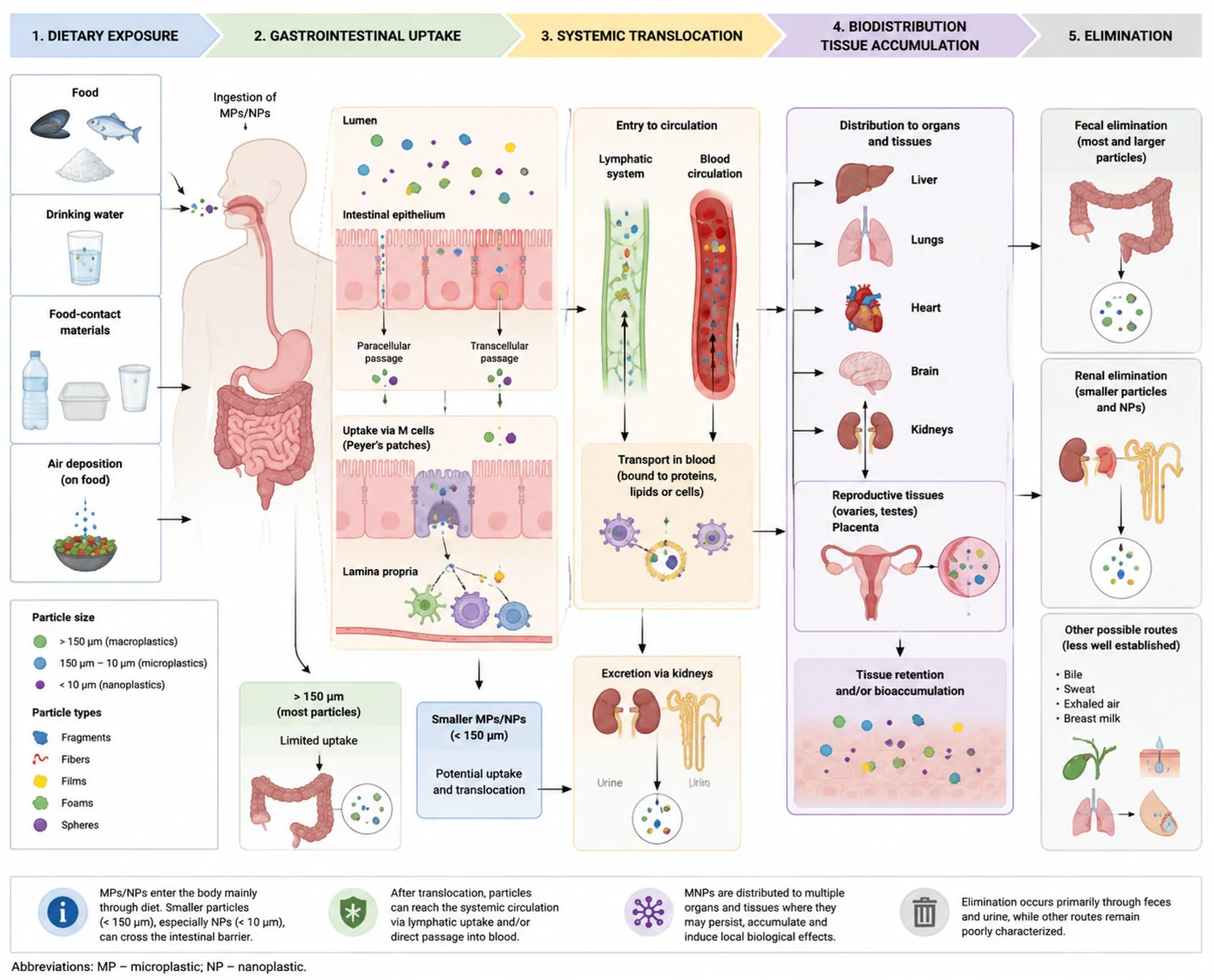
Schematic representation of the principal pathways of dietary exposure, gastrointestinal uptake, systemic translocation, biodistribution, tissue retention, and elimination of microplastics and nanoplastics.

The principal pathways of gastrointestinal uptake, systemic translocation, biodistribution, and elimination of MPs and NPs are illustrated in Figure 1.

### 1.4. Mechanisms of Toxicity

The toxicity of micro- and nanoplastics is multifactorial and may result from the particles themselves as well as from associated chemical substances and microorganisms transported on their surfaces [4,10,14]. Oxidative stress is considered one of the principal mechanisms underlying MNP toxicity and involves excessive production of reactive oxygen species (ROS), leading to oxidative damage to lipids, proteins, and DNA [4,10]. Inflammatory responses may develop through immune cell activation and increased secretion of pro-inflammatory cytokines, including IL-1β, IL-6, and TNF-α, potentially contributing to chronic inflammation and tissue fibrosis [4,10,14].

Mitochondrial dysfunction represents an important potential source of MNP-induced ROS. Disruption of the mitochondrial electron transport chain may promote electron leakage and the one-electron reduction of molecular oxygen, resulting in increased formation of superoxide anion radicals (O_2_•−) and subsequent ROS. This process may further impair mitochondrial function, reduce ATP production, and amplify oxidative stress. An additional mechanism may involve metal ions adsorbed onto MNP surfaces. Redox-active transition metals, particularly iron and copper, may participate in Fenton-type reactions and promote Haber–Weiss chemistry, facilitating the conversion of superoxide anion radicals and hydrogen peroxide into highly reactive hydroxyl radicals (•OH). Thus, the capacity of MNPs to carry adsorbed metal ions may potentially amplify ROS generation and oxidative damage. Apoptosis and autophagy may also become dysregulated through the activation of pathways involved in programmed cell death and disruption of cellular mechanisms responsible for the removal of damaged organelles [4,10,14].

Genotoxic effects include DNA damage, chromosomal instability, and an increased risk of mutations [4,10,14]. Alterations in immune function may involve both abnormal activation of immune cells and changes in the composition of the gut microbiota, potentially contributing to chronic inflammatory conditions and metabolic disturbances [4,6,10,14].

## 2. Effects of Microplastics and Nanoplastics on the Gastrointestinal System

The gastrointestinal tract represents the primary route of human exposure to microplastics (MPs) and nanoplastics (NPs) [11,34,35,36]. These particles enter the human body primarily through food and drinking water, as well as indirectly through the consumption of products contaminated during production, transportation, or storage [34,35,37,38,39,40]. Owing to the widespread presence of plastics in the environment, exposure is chronic and affects virtually the entire population [35,38,39,41,42,43]. It has been estimated that an average adult may ingest approximately 39,000–52,000 microplastic particles annually, while estimates considering all potential exposure sources have suggested that the total mass of plastic consumed could reach the equivalent of a credit card per week [11,36,44,45]. Although actual exposure levels vary depending on dietary patterns, place of residence, and drinking water quality, the gastrointestinal tract remains the principal site of contact between the human body and these contaminants [35,36,38,40]. The principal organ-specific effects of MNP exposure and the current strength of the supporting evidence are summarized in Table 3.

### 2.1. Absorption and Accumulation in the Gastrointestinal Tract

Following ingestion, gastrointestinal uptake of MNPs is strongly size-dependent. Particles in the larger micrometer range, particularly those above approximately 100–150 µm, are expected to remain predominantly within the gastrointestinal lumen and to be subsequently eliminated in feces, whereas smaller MPs (<10–20 µm) show a greater potential for interaction with and translocation across the intestinal epithelium. This potential increases further at the nanoscale (<1 µm), particularly for NPs below approximately 100 nm. Experimental evidence indicates that particles in the submicrometer and low-micrometer ranges can exhibit substantially greater cellular uptake than larger MPs. Overall, the available evidence therefore indicates an inverse relationship between particle size and the potential for systemic uptake and tissue accumulation. However, these size ranges should be regarded as approximate rather than absolute biological thresholds, because the available studies differ substantially in particle size, polymer type, surface properties, exposure dose, experimental model, and analytical methodology [34,36,39,43,46,47,48,49].

An increasing number of studies have confirmed the presence of microplastics in human biological samples, indicating their potential for long-term retention within the body [11,36,38,41]. These particles have been detected not only in feces but also in colon tissue, the liver, and other organs [11,37,49]. Particularly high concentrations have been reported in diseased liver tissue from patients with cirrhosis, suggesting that particle accumulation may increase with the progression of pathological processes [35,40,48]. This capacity for accumulation may increase the risk of chronic biological effects even under conditions of relatively low daily exposure [11,41,51].

### 2.2. Disruption of the Gut Microbiota

Experimental studies indicate that MNP exposure may induce gut microbiota dysbiosis, characterized by alterations in the relative abundance of beneficial and potentially pathogenic microorganisms [35,38,43]. Numerous experimental models have demonstrated reductions in bacteria belonging to the phylum Bacteroidota and in probiotic bacteria of the genus *Lactobacillus*, which are associated with anti-inflammatory activity and protection of the intestinal epithelium [35,36,52]. Concurrently, increases in bacteria belonging to the phyla Bacillota and Pseudomonadota, as well as potentially pathogenic members of the family Enterobacteriaceae, have been reported [35,38]. These alterations disrupt gastrointestinal homeostasis and may promote the development of chronic inflammation [35,36,52].

Dysbiosis is also associated with alterations in the metabolic activity of the gut microbiota [35,38]. Of particular importance is the reduced production of short-chain fatty acids (SCFAs), especially butyrate, which serves as a major energy source for colonocytes and contributes to the maintenance of intestinal barrier integrity [35,52]. Reduced SCFA production may impair the protective functions of the intestinal mucosa, intensify inflammatory processes, and increase epithelial susceptibility to injury [52]. An additional concern is the ability of microplastics to support the formation of microbial biofilms on their surfaces, collectively referred to as the plastisphere [36,45,52]. These biofilms provide a favorable environment for microbial colonization, including by potentially pathogenic bacteria such as *Escherichia coli*. Microplastics may therefore act as vectors facilitating the transport of potentially harmful microorganisms and their virulence factors into the gastrointestinal tract. *E. coli*, for example, has been reported to bind to microplastic surfaces and potentially deliver bacterial toxins to the colonic epithelium, which may contribute to epithelial injury and intestinal inflammation; however, direct evidence demonstrating an increased incidence of gastrointestinal infections in humans remains limited [36,52].

### 2.3. Impairment of the Intestinal Barrier

Microplastics and nanoplastics may also directly affect the structural integrity of the intestinal barrier [35,38,53]. One of the major mechanisms underlying their gastrointestinal toxicity involves disruption of tight junctions between intestinal epithelial cells [38,52]. Studies have demonstrated reduced expression of structural proteins such as zonula occludens-1 (ZO-1), occludin, and claudin-1, which are essential for maintaining epithelial integrity [38]. Their downregulation results in increased intestinal permeability, commonly referred to as a “leaky gut” [35,42]. Consequently, bacteria, bacterial toxins, and other potentially harmful substances present in the intestinal lumen may more readily enter the circulation, promoting both local and systemic inflammatory responses [35,38,53].

The chemical protective barrier of the intestine may also be adversely affected. Exposure to MPs and NPs has been associated with a reduction in the number of mucus-producing goblet cells and thinning of the mucus layer covering the intestinal epithelium [37,52]. Weakening of this natural protective barrier increases epithelial exposure to irritants and pathogens [11,38]. Consistent with the general mechanisms described in Section 1.4, experimental studies have demonstrated inflammatory cell infiltration, intestinal villus damage, and degenerative epithelial alterations that may impair nutrient absorption [35,37,38,41,44,53].

### 2.4. Gastrointestinal Diseases

A growing body of evidence suggests that the alterations described above may contribute to the pathogenesis or progression of gastrointestinal diseases [38,40]. Particular attention has been directed toward the association between microplastic exposure and inflammatory bowel disease (IBD), including ulcerative colitis and Crohn’s disease [38,54]. Patients with these conditions have been reported to exhibit higher concentrations of microplastics in feces than healthy individuals, with particle abundance positively correlated with disease severity [38,52]. Although these observations indicate an association between MNP burden and IBD severity, they do not establish whether MNP exposure contributes to disease development or progression, or whether gastrointestinal pathology itself promotes increased particle retention [38,40,43].

MNPs have also been investigated in relation to colorectal cancer. Higher particle concentrations have been reported in colorectal tumor tissues than in adjacent non-tumorous tissues; however, these observations do not establish temporality or causality and may reflect altered particle retention in diseased tissue [37,38,49]. The potential carcinogenic relevance of MNP exposure and the limitations of the available evidence are discussed in detail in Section 9.

The potential effects of microplastics extend beyond the gastrointestinal tract itself [35,38,42]. Disruption of the gut–liver axis may contribute to the development of metabolic dysfunction-associated steatotic liver disease (MASLD), formerly referred to as non-alcoholic fatty liver disease (NAFLD) [35,37]. Experimental evidence suggests that disruption of the gut–liver axis by MNPs may be accompanied by inflammatory responses and alterations in lipid and glucose metabolism [52,55,56]. However, whether these changes translate into an increased risk of insulin resistance, type 2 diabetes, or obesity in humans remains unclear [35,51]. Adverse effects on pancreatic function and inhibition of digestive enzymes, including pepsin and α-amylase, have also been reported, which may impair digestive efficiency and nutrient utilization [34,38,55].

## 3. Effects of Microplastics and Nanoplastics on the Respiratory System

Although the gastrointestinal tract represents the principal route of exposure to microplastics (MPs) and nanoplastics (NPs) in the context of the human diet, the respiratory system constitutes an important additional route of exposure and a potential target of their systemic toxicity [57,58,59,60]. Unlike dietary exposure, which occurs primarily through contaminated food and drinking water, respiratory exposure results from the continuous inhalation of airborne plastic particles [58,60,61]. Micro- and nanoplastics have been detected in both outdoor and indoor environments, with substantially higher concentrations frequently reported in enclosed spaces [58,60,62]. Major sources include fibers released from synthetic textiles, household dust, building materials, furniture and other indoor furnishings, as well as particles generated by tire and brake wear [58,60,61,63]. Owing to the direct contact of the respiratory epithelium with inhaled air, the lungs represent an important target organ for these contaminants [57,61,64,65].

Recent studies have demonstrated the presence of microplastics not only in airborne samples but also in sputum, lung tissue, bronchoalveolar lavage fluid, and specimens collected during surgical procedures [58,66,67]. These findings indicate that inhaled particles may not be completely removed by respiratory clearance mechanisms and can persist in lung tissue, potentially contributing to chronic pathological processes [57,63,65].

### 3.1. Inhalation Exposure and Particle Deposition

Inhalation represents an important complementary source of human exposure to micro- and nanoplastics alongside dietary intake [57,58,59]. It has been estimated that an adult may inhale approximately 3000 to 68,000 plastic particles per day, although actual exposure levels vary considerably depending on the environment, air quality, lifestyle, and other environmental conditions [58,63,68]. The highest concentrations are generally observed indoors, where synthetic fibers released from clothing, carpets, upholstered furniture, and other household materials constitute major sources of airborne microplastics [58,60,62]. Additional contributions arise from tire wear particles, building materials, and industrial dust [11,60,61].

Following inhalation, the site of particle deposition is determined primarily by aerodynamic equivalent diameter (AED), shape, density, and surface properties [58,59,63,69]. Three principal physical mechanisms govern deposition: Brownian diffusion, gravitational sedimentation, and inertial impaction [59,64,70]. Particles measuring 5–10 µm are predominantly retained in the nasal cavity and pharynx, where they may be partially removed by mucociliary clearance [59,61,64]. Fractions measuring 2–5 µm can reach the trachea and bronchi, whereas particles smaller than 1 µm may penetrate into the alveolar region [58,61]. The smallest nanoplastics, particularly those below 100 nm, may cross the alveolar–capillary barrier, potentially gaining access to the systemic circulation and lymphatic system and subsequently reaching distant organs [65,70].

Particle shape also substantially influences respiratory deposition and retention [57,59,69,71]. Synthetic fibers exhibit aerodynamic properties distinct from those of spherical particles and may therefore penetrate peripheral regions of the lungs and remain within pulmonary tissue for prolonged periods [57,58,69]. Their elongated morphology may impair complete phagocytosis by alveolar macrophages, thereby promoting persistent inflammatory responses [63,69]. Deposition is additionally influenced by breathing patterns. Rapid, shallow breathing favors the deposition of larger particles in the upper respiratory tract, whereas slower and deeper breathing may facilitate the transport of smaller particles into the distal lung regions [57,59].

Despite respiratory defense mechanisms, including mucociliary clearance, coughing, and alveolar macrophage activity, a fraction of inhaled MPs and NPs may persist in lung tissue [61,69]. Chronic particle retention may contribute to sustained local inflammation and increase the likelihood of progressive pathological alterations [57,63,69].

### 3.2. Impairment of Pulmonary Function

Exposure to micro- and nanoplastics may adversely affect normal pulmonary function through multiple interacting mechanisms [59,61,66]. Of particular importance is the interaction of plastic particles with pulmonary surfactant, a complex mixture of phospholipids and proteins lining the alveolar surface [57]. Experimental evidence suggests that microplastics can interact with surfactant components, alter their biophysical properties, and increase surface tension [58,68]. These alterations may reduce alveolar stability, impair gas exchange, and potentially increase susceptibility to alveolar collapse [59,68].

Exposure to airborne microplastics has also been associated with alterations in pulmonary function parameters [11,60,66]. In populations exposed to high concentrations of synthetic microfibers, reductions in forced expiratory volume in one second (FEV_1_) and forced vital capacity (FVC) have been reported, suggesting impaired ventilatory function [63,68]. Although the mechanisms underlying these observations remain incompletely understood, chronic inflammation, airway remodeling, and respiratory epithelial injury may contribute to these functional abnormalities [57,61,66].

Micro- and nanoplastics may also promote airway hyperresponsiveness, resulting in an exaggerated response to respiratory irritants [57,58,65]. Potential consequences include bronchial smooth muscle contraction, increased mucus secretion, and airflow limitation [58,65]. These effects appear to be associated with immune cell activation and increased production of pro-inflammatory mediators [57,68].

MNP-associated oxidative stress and mitochondrial dysfunction may contribute to respiratory epithelial injury and cell death [57,61,70,71]. In addition, disruption of intercellular junctions may increase epithelial barrier permeability, potentially facilitating the penetration of pathogens, allergens, and other environmental contaminants into deeper pulmonary tissues [58,65,68].

### 3.3. Respiratory Diseases

Chronic exposure to micro- and nanoplastics has increasingly been investigated as a potential contributor to respiratory disease development and progression [58,61,71]. Particular attention has been directed toward bronchial asthma [58,61,65,71]. Experimental studies indicate that plastic particles may enhance Th2-mediated immune responses, increase the production of pro-allergic interleukins, promote infiltration by eosinophils, macrophages, and lymphocytes, and stimulate mucus production by goblet cells [57,68]. These effects may intensify airway inflammation and hyperresponsiveness, potentially contributing to more severe asthma manifestations [61,65].

Experimental evidence suggests that MNP exposure may promote pathological processes relevant to chronic obstructive pulmonary disease (COPD), including endoplasmic reticulum stress, alveolar injury, and airway remodeling [11,58,61,71]. However, direct evidence linking MNP exposure to COPD development or progression in humans remains limited.

Pulmonary fibrosis represents another potential consequence of sustained inflammatory responses to MPs and NPs [11,61]. Experimental evidence indicates that these particles may stimulate fibroblast activation and excessive deposition of collagen and other extracellular matrix components [57,58]. Progressive remodeling of the pulmonary parenchyma may subsequently reduce tissue elasticity and decrease the effective surface area available for gas exchange [57,59]. Such mechanisms could potentially contribute to interstitial lung abnormalities and persistent impairment of respiratory function [58,71].

The potential involvement of microplastics in pulmonary carcinogenesis has also attracted increasing attention [58,71]. Plastic particles have been detected in ground-glass nodules (GGNs), some of which may represent early-stage pulmonary neoplastic lesions [69]. The previously described inflammatory, oxidative, and genotoxic effects of MNPs, together with their capacity to carry adsorbed toxicants, provide biologically plausible mechanisms through which these particles could influence carcinogenic processes [57,71]. However, current evidence is insufficient to establish a causal relationship between MNP exposure and lung cancer in humans, and this potential association requires confirmation in well-designed epidemiological and mechanistic studies [58,64,66].

Microplastics may additionally increase susceptibility to respiratory infections [57,70]. Damage to the respiratory epithelial barrier and impairment of alveolar macrophage function may weaken local immune defenses and facilitate microbial colonization of the respiratory tract [70,71]. Moreover, microplastic surfaces can support the adsorption and transport of microorganisms and microbial products, potentially allowing these particles to act as vectors for biological contaminants [61,66]. It has been proposed that airborne plastic particles may participate in the transport of viruses, including SARS-CoV-2 and influenza A virus (H1N1); however, the relevance of this mechanism under real-world exposure conditions remains uncertain and requires further investigation [60,71].

## 4. Effects of Microplastics and Nanoplastics on the Cardiovascular System

The cardiovascular system plays a central role in the systemic distribution of substances present within the body. Once microplastics (MPs) and nanoplastics (NPs) enter the bloodstream, they may be transported to multiple organs and tissues [72,73,74]. Although MNPs were previously thought to remain largely confined to their site of entry, recent evidence indicates that particularly small particles can cross biological barriers, persist in the circulation, and accumulate in internal organs [72,75]. Growing evidence further suggests that circulating MNPs are not biologically inert but may interact directly with cardiovascular cells and tissues, promoting oxidative stress, chronic inflammation, endothelial dysfunction, and myocardial injury [72]. These findings have led to increasing interest in MNPs as emerging environmental contributors to cardiovascular risk [72,75,76].

In the context of dietary exposure, the cardiovascular system is particularly relevant because particles that cross the intestinal barrier may subsequently enter the lymphatic and systemic circulation and reach distant organs. Thus, the transition from gastrointestinal exposure to systemic distribution represents a critical step linking the ingestion of MNPs with potential cardiovascular effects.

### 4.1. Entry into the Circulation and Systemic Distribution

Micro- and nanoplastics enter the human body primarily through the gastrointestinal and respiratory routes, whereas dermal absorption is generally considered less significant [72,74]. Following ingestion, plastic particles may cross the intestinal barrier and enter the lymphatic system and systemic circulation [77,78,79]. Proposed mechanisms include transcytosis through enterocytes and microfold (M) cells within Peyer’s patches, as well as passage through intercellular spaces in a process described as persorption [77,78].

The capacity for gastrointestinal translocation is strongly influenced by particle size. Nanoplastics, particularly those smaller than 50 nm, generally exhibit greater bioavailability than larger microplastic particles and may more readily reach the systemic circulation [72,77,78]. This size-dependent translocation is particularly relevant to dietary exposure because it determines the fraction of ingested particles capable of reaching the cardiovascular system and other distant organs.

Inhalation represents an additional route through which MNPs may enter the circulation [75,80]. Small particles reaching the pulmonary alveoli may cross the alveolar–capillary barrier and enter the pulmonary microvasculature [78,79]. Once present in the bloodstream, particles may be transported to highly perfused organs, including the heart, brain, liver, and kidneys [73,75,78]. Dermal uptake through intact skin appears limited, although nanoplastics may potentially enter through hair follicles, sweat glands, or damaged epidermis [72,74,78].

The presence of microplastics in human blood has now been directly demonstrated [71,76,81]. Polyethylene (PE), polyethylene terephthalate (PET), polystyrene (PS), and other commonly used polymers have been identified in venous blood samples [76,81]. Some studies have reported detectable microplastics in approximately 77–80% of examined individuals, with mean concentrations of approximately 1.6 µg/mL [72,76,81]. These observations suggest that chronic environmental and dietary exposure may result in the persistent presence of plastic particles within the systemic circulation [72,73].

Following entry into plasma, the surfaces of nanoplastics may rapidly become coated with proteins, lipids, lipoproteins, and other biomolecules [72,74,82]. The resulting biomolecular or protein corona modifies particle physicochemical properties and may influence immune recognition, circulation time, cellular uptake, and organ distribution [72,74]. The composition of the corona may also alter MNP toxicity by modifying interactions with endothelial cells, macrophages, and cardiomyocytes [72,74,79].

### 4.2. Endothelial Dysfunction and Vascular Injury

The vascular endothelium represents one of the first cardiovascular structures directly exposed to circulating micro- and nanoplastics [72,74]. This cell layer regulates vascular permeability, inflammatory responses, coagulation, and vascular tone, particularly through the production of nitric oxide (NO). Endothelial dysfunction is widely recognized as an early event in the development of cardiovascular disease [71,72].

Experimental studies indicate that MNP exposure may reduce endothelial cell viability and disrupt intercellular junctions responsible for maintaining vascular barrier integrity. Reduced expression of tight-junction proteins, including ZO-1, has been associated with increased vascular permeability [71,79]. Such alterations may facilitate the migration of inflammatory cells and other potentially damaging factors into the vascular wall, thereby promoting chronic vascular inflammation [72,75].

MNP-associated oxidative and inflammatory responses may contribute to endothelial dysfunction, including activation of NF-κB and MAPK signaling and increased expression of the adhesion molecules VCAM-1 and ICAM-1 [72,73,75,77,78]. These endothelial alterations may promote leukocyte adhesion and vascular inflammation, providing a plausible mechanistic link between MNP exposure and processes involved in atherogenesis [71,72,75].

MNP exposure may also accelerate endothelial cellular senescence [72,77,83]. Prolonged exposure has been associated with reduced proliferative capacity and impaired vascular repair mechanisms [71,72,82]. Angiogenic capacity may also be impaired through disruption of vascular endothelial growth factor (VEGF)-dependent signaling [74,82].

Another important consequence is reduced nitric oxide bioavailability [71,72]. Decreased NO production shifts vascular regulation toward vasoconstriction, increasing vascular resistance and further impairing endothelial function [72,74]. Taken together, these mechanisms provide a plausible biological pathway through which systemically distributed MNPs may contribute to vascular dysfunction after chronic exposure.

### 4.3. Cardiovascular Diseases

A growing body of evidence suggests that the chronic presence of micro- and nanoplastics in the circulation may contribute to the development or progression of cardiovascular disease [72]. Particular attention has been directed toward atherosclerosis [72,75]. Microplastic particles have been identified in carotid and coronary atherosclerotic plaques, with polyethylene and polyvinyl chloride (PVC) among the most frequently detected polymers [71,72,76].

Their presence has been associated with increased local inflammation, macrophage activation, and enhanced formation of foam cells involved in plaque progression [71,72,83]. MNPs may also reduce plaque stability by promoting extracellular matrix degradation and increasing proteolytic activity [71,72].

The potential clinical relevance of these observations was highlighted in patients undergoing carotid endarterectomy. The presence of micro- and nanoplastics within atherosclerotic plaques was associated with an approximately 4.5-fold higher risk of myocardial infarction, stroke, or cardiovascular death during approximately 34 months of follow-up [4,72,76,84]. Importantly, this observational association does not demonstrate that MNPs caused the reported cardiovascular events, as residual confounding, differences in cumulative exposure, and characteristics associated with pre-existing cardiovascular disease cannot be excluded. Nevertheless, these findings provide clinically relevant evidence supporting further investigation of MNPs as a potential environmental factor associated with cardiovascular risk [4,84].

MNPs may also interact directly with circulating blood cells. Experimental studies have demonstrated damage to erythrocyte membranes, alterations in cell morphology, and hemolysis following exposure to plastic particles [74,76,77]. Increased platelet activation and aggregation have also been reported, potentially promoting coagulation and thrombus formation [75,79,82]. These effects may theoretically increase susceptibility to acute cardiovascular events, including thromboembolism, myocardial infarction, and ischemic stroke [74,76].

MNPs have also been investigated for their potential effects on the myocardium. Plastic particles have been detected in myocardial tissue, the pericardium, and other cardiovascular structures [74,75,76]. Experimental studies indicate that MNP exposure may disrupt mitochondrial function and calcium homeostasis in cardiomyocytes, with potential consequences for ATP production, contractility, and electrical conduction [71,72,73,75,78,82,85].

Chronic exposure may additionally promote structural remodeling of cardiac tissue. Experimental studies have reported myocardial fibrosis, cardiomyocyte hypertrophy, and ventricular wall remodeling following prolonged MNP exposure [72,74,75]. These changes may impair both systolic and diastolic function and increase susceptibility to arrhythmias, including bradycardia and other conduction abnormalities [72,75]. In animal models, chronic exposure has also been associated with reduced left ventricular ejection fraction, chamber dilation, and features consistent with progressive heart failure [4,73,75].

## 5. Effects of Microplastics and Nanoplastics on the Nervous System

The nervous system is particularly vulnerable to environmental stressors because its proper function depends on stable cellular homeostasis, efficient synaptic communication, and high metabolic activity [86,87]. Microplastics (MPs) and nanoplastics (NPs) are increasingly being investigated as potential neurotoxic contaminants capable of inducing oxidative stress, chronic neuroinflammation, mitochondrial dysfunction, neurotransmission disturbances, and structural cellular damage [86,88]. Nanoplastics are considered particularly relevant because their small size, high specific surface area, and capacity to cross biological barriers may facilitate access to the central nervous system and direct interactions with neurons and glial cells [4,86].

In the context of dietary exposure, neurological effects may arise after gastrointestinal uptake, systemic circulation, and subsequent translocation of sufficiently small particles across the blood–brain barrier. In addition, intestinal dysbiosis and disruption of the gut–brain axis may provide an indirect pathway through which ingested MNPs influence central nervous system function.

### 5.1. Translocation to the Central Nervous System

The blood–brain barrier (BBB) provides essential protection to the central nervous system by restricting the entry of toxins, pathogens, and foreign particles from the circulation into brain tissue [86,89]. It is formed primarily by brain microvascular endothelial cells connected by tight junctions, together with pericytes, astrocytes, and the basement membrane [89,90,91]. Despite its high selectivity, experimental studies indicate that MNPs, particularly particles smaller than 100 nm, may cross the BBB and accumulate within brain structures [86,92].

Transport across the BBB may occur through several mechanisms, including transcytosis, clathrin- or caveolin-mediated endocytosis, micropinocytosis, and paracellular passage following disruption of tight-junction integrity [86,92]. Owing to their small dimensions, nanoplastics may be internalized by endothelial cells and subsequently transported toward the brain-facing side of the vascular barrier [4,86,88]. At the same time, MNP exposure may impair BBB integrity by reducing the expression of tight-junction proteins such as occludin, claudin-5, and ZO-1 [4,86]. Increased barrier permeability may in turn facilitate the entry of inflammatory mediators, toxicants, and additional plastic particles into the central nervous system [86,88].

An additional factor influencing particle behavior is the formation of a biomolecular corona following contact with blood and biological fluids [4,86]. Proteins, lipids, and other biomolecules adsorb onto the particle surface, creating a new biological identity that may affect cellular recognition, circulation time, and the ability to cross biological barriers [4,86]. The presence of cholesterol and selected lipids within the corona may increase nanoplastic affinity for cellular membranes and facilitate interaction with BBB components, whereas some protein coatings may reduce translocation [4,86,93].

MNPs may also reach or affect the nervous system through alternative pathways [86,89,91,94,95]. The nose-to-brain pathway may allow inhaled particles deposited on the olfactory epithelium to reach the olfactory bulb and other brain regions along neuronal pathways [86,91,94]. From the perspective of dietary exposure, however, particular importance should be attributed to the gut–brain axis. MNP-induced dysbiosis, intestinal inflammation, and altered vagal signaling may influence brain function indirectly even without substantial direct particle translocation [4,86,87]. BBB integrity may also be weakened by aging, infection, chronic inflammatory conditions, and oxidative stress, potentially increasing central nervous system susceptibility to MNP accumulation [86,88,91].

### 5.2. Neuroinflammation, Oxidative Stress, and Functional Impairment

Following entry into nervous tissue, MNPs may accumulate in brain regions involved in cognitive, emotional, and motor functions, including the hippocampus, prefrontal cortex, cerebellum, and limbic structures [91,95,96,97]. Their presence may interfere with neuronal communication, synaptic integrity, glial activity, and neural plasticity [4,89,93].

Consistent with the general mechanisms described in Section 1.4, experimental studies indicate that MNP-associated neurotoxicity involves interconnected inflammatory, oxidative, and mitochondrial responses. In nervous tissue, these effects are particularly associated with microglial activation, neuroinflammatory signaling, impaired mitochondrial function, and subsequent neuronal injury [4,86,87,88,89,92,93,94,95,96,97,98,99,100].

MNP exposure may also disrupt neurotransmission [91,97]. Alterations have been reported in the synthesis, release, and reuptake of neurotransmitters, including dopamine, serotonin, acetylcholine, GABA, and glutamate [91,92,95]. Inhibition of acetylcholinesterase activity has been frequently described and may impair cholinergic signaling [89,93,94,99]. These disturbances may affect learning, memory, attention, and motor control. Disruption of the balance between excitatory and inhibitory neurotransmission may also contribute to neuronal hyperexcitability, mood disturbances, and cognitive deficits [86,87,95].

Experimental studies have associated chronic MNP exposure with impaired learning and memory, anxiety- and depression-like behavior, motor deficits, and alterations in social behavior [86,87]. Particular concern has been raised regarding prenatal and early-life exposure [86,99]. MNPs may cross the placental barrier and potentially reach the developing fetal brain [87,99]. Experimental findings suggest possible disturbances in neuronal migration, synaptogenesis, cortical development, and maturation of neural networks [4,91]. Accordingly, maternal exposure has been investigated as a potential risk factor for neurodevelopmental disturbances, although evidence in humans remains limited [93,99].

### 5.3. Potential Neurodegenerative Effects

A growing body of experimental evidence suggests that MNPs may influence mechanisms involved in the initiation or progression of neurodegenerative diseases [86,88]. Particular attention has been given to their potential effects on protein misfolding and aggregation, processes central to the pathogenesis of Alzheimer’s disease, Parkinson’s disease, amyotrophic lateral sclerosis, and other neurodegenerative disorders [88].

In the context of Alzheimer’s disease, nanoplastics have been investigated for their potential interactions with amyloid-β and tau proteins [86,91,92]. Their surfaces may provide interfaces that facilitate protein adsorption, abnormal folding, and aggregation, thereby potentially accelerating the formation of toxic oligomers [86,94]. MNPs may also interfere with microglial clearance of amyloid species, thereby promoting chronic inflammation and synaptic injury [86,99]. These mechanisms could theoretically contribute to cognitive decline, although current evidence does not establish a causal role of MNP exposure in human Alzheimer’s disease [86,92].

In Parkinson’s disease, particular attention has been directed toward α-synuclein aggregation and dopaminergic neuron vulnerability [86,92,93]. Experimental studies suggest that selected nanoplastics, including polystyrene and polyethylene particles, may promote α-synuclein aggregation and increase toxicity toward dopaminergic neurons in the substantia nigra [88,91]. Such mechanisms could disrupt dopaminergic neurotransmission and potentially contribute to motor abnormalities [86,99].

MNPs have also been discussed in relation to amyotrophic lateral sclerosis. Experimental findings have linked nanoplastic exposure with TDP-43 aggregation, a pathological feature associated with ALS [4,86,87]. Potential effects on myelination and demyelinating processes have also been proposed in the context of multiple sclerosis [86,99]. However, these associations remain largely mechanistic or experimental and should not be interpreted as evidence that MNP exposure directly causes these diseases.

The detection of microplastics in human brain tissue has further intensified interest in their potential neurological significance [88,91]. Post-mortem studies have suggested that plastic particle concentrations may be higher in brains from individuals with dementia than in those without diagnosed neurodegenerative disease, with polyethylene among the most frequently identified polymers [4,86,87]. These findings do not establish causality but reinforce the need for further investigation into long-term MNP accumulation and neurological outcomes [99].

Experimental evidence further suggests that MNP exposure may impair neural repair and neuroplasticity [4,86]. These effects could theoretically be relevant to neurological recovery and neurodegenerative processes; however, their clinical significance remains uncertain and requires confirmation in dedicated human studies [86,87,97].

## 6. Effects of Microplastics and Nanoplastics on the Urinary System

The urinary system, particularly the kidneys, represents a potential target of the adverse effects of microplastics (MPs) and nanoplastics (NPs) [38,101,102,103]. This vulnerability is closely related to the fundamental role of the kidneys in blood filtration, the removal of metabolic waste products, and the elimination of xenobiotics [40,101,103]. Following their absorption, including through the gastrointestinal tract after dietary exposure, and subsequent entry into the systemic circulation, plastic particles may be transported to the renal parenchyma, where they interact with glomerular cells, tubular epithelial cells, and interstitial structures [40,103]. Nanoplastics are of particular concern because their small size facilitates their passage across biological barriers and enables their internalization into cells and subcellular organelles [25,103,104].

### 6.1. Accumulation in the Kidneys

The accumulation of micro- and nanoplastics in the kidneys has been demonstrated primarily in experimental studies, while emerging evidence also indicates their presence in human biological samples [38,101,105]. Plastic particles have been detected in urine samples and renal tissues using, among other approaches, spectroscopic techniques capable of identifying polymers such as polyethylene, polystyrene, and polyethylene terephthalate [40,104]. Their presence in urine suggests that at least a fraction of absorbed MNPs may undergo renal filtration or urinary excretion, whereas their detection in renal tissue indicates the possibility of retention within the renal parenchyma [40,102,103].

The extent of renal accumulation is influenced by particle size and physicochemical characteristics. Smaller particles, particularly NPs, more readily cross biological membranes, enter the systemic circulation, and reach renal tissue. Sufficiently small particles may also pass through the glomerular filtration barrier and subsequently be taken up by proximal tubular epithelial cells through endocytic or pinocytic mechanisms [101,102]. Particle shape and surface charge can further influence interactions with cellular membranes and the efficiency of cellular uptake, whereas polymer composition, hydrophobicity, and surface reactivity may modify interactions with biological components and thereby affect renal retention and toxicity [101]. Nanoplastics cross tissue barriers more readily than larger microplastics and may therefore reach higher intracellular concentrations in renal cells [102,104]. They may accumulate not only within the extracellular compartment but also inside lysosomes and mitochondria, thereby increasing the risk of metabolic dysfunction and cellular injury [40,104]. Within the kidney, plastic particles may localize in the glomeruli, mesangial cells, capillary structures, and proximal tubular epithelium, which is particularly susceptible to toxic injury because of its high metabolic activity [40,101,102].

Prenatal exposure may also have important implications [38,101]. Animal studies suggest that nanoplastics can cross the placental barrier and accumulate in the developing fetal kidneys [40,101]. Such exposure may interfere with nephrogenesis, including nephron formation, and potentially increase susceptibility to renal injury later in life [38,101].

### 6.2. Impairment of Renal Function

The presence and accumulation of micro- and nanoplastics in the kidneys may impair their fundamental filtration, metabolic, and regulatory functions [101,102,103,106]. In experimental studies, exposure to MNPs has been associated with alterations in serum creatinine, urea, blood urea nitrogen, and uric acid levels, which are commonly used as indicators of renal function [38,40]. Increased creatinine and urea levels have been frequently reported, potentially reflecting impaired glomerular filtration and a reduced capacity of the kidneys to eliminate nitrogenous metabolic waste products [101,104].

Micro- and nanoplastics may also damage the glomerular filtration barrier [102,103,106]. Of particular importance is their effect on podocytes, which are essential for maintaining the selectivity of glomerular filtration [103]. Podocyte injury may increase urinary protein loss, potentially resulting in albuminuria or proteinuria [102,105]. An association between the presence of plastic polymers in urine and an increased urinary albumin-to-creatinine ratio has also been reported, suggesting a possible relationship between MNP exposure and early renal injury [105].

Another important consequence is the disruption of cellular energy metabolism [25,38,107]. Proximal tubular epithelial cells have exceptionally high ATP requirements; consequently, mitochondrial injury induced by nanoplastics may disrupt glycolysis, the tricarboxylic acid cycle, and ion transport [38,107,108]. These alterations may impair tubular reabsorption, disturb fluid and electrolyte balance, and compromise the ability of the kidneys to maintain systemic homeostasis [101,106]. Some studies further suggest that chronic renal accumulation of MNPs may indirectly contribute to the development of hypertension through disturbances in renal vascular and regulatory functions [23,25,38,109].

### 6.3. Nephrotoxicity

Consistent with the general mechanisms described in Section 1.4, experimental studies indicate that renal MNP toxicity involves interconnected oxidative and inflammatory responses that may contribute to mitochondrial and DNA damage, tubular injury, inflammatory cell infiltration, and renal tissue remodeling [38,40,101,102,103,106,107,108,110]. Concurrently, endoplasmic reticulum stress and the activation of apoptosis, autophagy, necroptosis, and ferroptosis have been reported. Ferroptosis, an iron-dependent form of cell death driven by lipid peroxidation, may be particularly relevant to the development and progression of renal fibrosis [38,104,107].

Histopathological alterations associated with MNP exposure include tubular atrophy and necrosis, glomerular collapse, thickening of the basement membrane, and inflammatory infiltration of the renal interstitium [38,102]. Chronic exposure may additionally promote epithelial–mesenchymal transition and excessive collagen deposition, ultimately resulting in renal fibrosis [103,104]. This process is of particular clinical relevance because fibrosis is a major pathological feature of chronic kidney disease and is generally associated with the irreversible loss of functional renal parenchyma [101,102].

The previously described carrier function of MNPs may additionally facilitate renal exposure to adsorbed nephrotoxicants, including cadmium, lead, arsenic, bisphenol A, and phthalates [23,102,104,110,111]. The gut–kidney axis may also contribute to renal toxicity following dietary exposure, as MNP-induced disruption of the intestinal barrier may facilitate the systemic translocation of endotoxins and inflammatory mediators [40,106].

## 7. Effects of Microplastics and Nanoplastics on the Reproductive System

The reproductive system is particularly susceptible to the adverse effects of microplastics (MPs) and nanoplastics (NPs), as its proper function depends on precise hormonal regulation, the integrity of biological barriers, and the maintenance of high-quality reproductive cells [112,113,114]. MNPs can enter the human body through ingestion, inhalation, and dermal contact, although dietary exposure represents one of the major routes of continuous population exposure. Following absorption and entry into the systemic circulation, these particles may reach reproductive organs and tissues [112,114,115,116]. Plastic-associated chemicals, including bisphenol A, phthalates, and persistent organic pollutants, may additionally contribute to reproductive toxicity through endocrine-disrupting effects [112,117,118]. The reproductive toxicity of MNPs is primarily associated with oxidative stress, chronic inflammation, DNA damage, impaired steroidogenesis, and abnormalities in gamete development and maturation [112,113,119].

### 7.1. Accumulation in Reproductive Organs

Available evidence indicates that micro- and nanoplastics can cross biological barriers associated with the reproductive system, including the blood–testis barrier, blood–follicle barrier, and placental barrier [115,116,120]. Their presence has been detected in the testes, semen, ovaries, follicular fluid, endometrium, uterus, placenta, breast milk, amniotic fluid, and neonatal meconium [112,116,121,122,123]. These findings indicate that absorbed plastic particles may undergo systemic distribution and reach structures directly involved in reproduction and embryonic and fetal development [112,117,124].

In males, MNPs have been identified in testicular tissue and semen [112,113,121]. The predominant polymers include polyethylene, polyvinyl chloride, polystyrene, polyethylene terephthalate, and nylon [112,113]. Their presence in the testes is of particular concern because these particles may alter the microenvironment of the seminiferous tubules, impair Sertoli and Leydig cell function, and disrupt spermatogenesis [112,125]. In females, microplastics have been detected in ovarian follicular fluid, suggesting the possibility of direct interactions between plastic particles and oocytes or granulosa cells [113,122]. MNPs may also accumulate in the endometrium, fallopian tubes, and uterus, which are essential for fertilization, embryo transport, and implantation [113,115].

Of particular concern is the detection of microplastics in the placenta [117,118]. Plastic particles have been identified on both the maternal and fetal sides of the placenta, suggesting their potential to cross the maternal–fetal interface [60,117,124]. Furthermore, the detection of MNPs in amniotic fluid, breast milk, and meconium indicates that exposure may begin during prenatal development and continue throughout the perinatal period [116,126].

### 7.2. Impairment of Fertility

Exposure to micro- and nanoplastics may adversely affect fertility in both males and females [116,120,127]. In males, one of the major mechanisms involves disruption of the blood–testis barrier, which protects developing germ cells from toxic substances and uncontrolled immune responses [112,113]. Loss of barrier integrity may result in seminiferous tubule degeneration, germ cell exfoliation, and impaired spermatogenesis [125,127,128]. Consequently, experimental studies, primarily conducted in rodent models, have associated MNP exposure with reduced sperm counts and motility, an increased proportion of morphologically abnormal spermatozoa, and greater sperm DNA fragmentation [11,118].

MNPs may also impair Leydig and Sertoli cell function [112,128]. Leydig cell dysfunction can reduce testosterone synthesis, whereas impairment of Sertoli cells may compromise the nutritional support and maturation of developing germ cells [112,127]. In addition, plastic particles and associated endocrine-disrupting chemicals may interfere with the hypothalamic–pituitary–testicular axis, altering luteinizing hormone (LH) and follicle-stimulating hormone (FSH) secretion [113,125]. These alterations may contribute to impaired semen quality and reduced male reproductive potential [112,113].

In females, experimental evidence suggests that MNP exposure may impair ovarian function, follicular development, and oocyte quality [116,118,129]. Reported alterations include oxidative stress and apoptosis in granulosa cells, increased follicular atresia, and potential reductions in ovarian reserve [116,122,124,127]. However, the extent to which these effects occur under typical human exposure conditions remains uncertain. Disrupted steroidogenesis may additionally result in altered estradiol and progesterone levels, menstrual cycle irregularities, and impaired conditions required for successful fertilization and implantation [116,118,122].

The uterus represents another potential target of MNP toxicity [118,124]. Plastic particles may induce inflammation, fibrosis, endometrial thinning, and a reduction in the number of uterine glands [116,119]. Such alterations may reduce endometrial receptivity and interfere with embryo implantation [116,118]. Consequently, exposure to micro- and nanoplastics may potentially contribute to infertility, implantation failure, and early pregnancy loss [115,116,118].

### 7.3. Effects on Embryonic and Fetal Development

The potential effects of micro- and nanoplastics on embryonic and fetal development are primarily associated with their ability to cross the placental barrier and impair placental function [117,118]. The placenta is responsible for the exchange of oxygen, nutrients, hormones, and metabolites between the maternal and fetal circulations [114,117,123]. Accumulation of MNPs within placental structures may induce oxidative stress, inflammatory responses, trophoblast injury, and impaired placental vascularization [116,117]. These alterations may compromise oxygen and nutrient transfer, potentially increasing the risk of intrauterine growth restriction, low birth weight, and other pregnancy complications [116,118].

Prenatal exposure to MNPs may also directly affect developing fetal organs [116,117,118]. Owing to their small size and high biological mobility, nanoplastics may reach the fetal liver, kidneys, heart, lungs, and brain, where they can interfere with cellular differentiation, energy metabolism, and oxidative–inflammatory homeostasis [116,118]. Experimental studies have reported reduced birth weight, altered organ development, metabolic abnormalities, and disturbances in hormonal regulation following prenatal MNP exposure [4,115,117].

The developing nervous system may be particularly susceptible to prenatal exposure [116,124]. Micro- and nanoplastics may interfere with neuronal maturation, synapse formation, and the organization of neuronal networks [116]. Animal studies have reported impaired cognitive function, motor deficits, increased anxiety-like behavior, and alterations in social behavior in offspring following prenatal exposure [116,124]. These findings suggest that the consequences of exposure may become apparent not only immediately after birth but also during later stages of development [117,118].

The possibility of transgenerational effects is also receiving increasing attention [113,116,118]. Micro- and nanoplastics may induce epigenetic alterations, including changes in DNA methylation, histone modifications, and microRNA expression [118,124]. Such mechanisms could potentially affect germ cell function and the development of subsequent generations, including offspring that were not directly exposed to plastic particles [112,118]. Although this field requires substantially more research, current evidence suggests that the reproductive and developmental effects of MNP exposure may extend beyond directly exposed individuals and potentially affect subsequent generations [116,117].

## 8. Effects of Microplastics and Nanoplastics on Endocrine Homeostasis

The endocrine system is one of the major regulatory networks of the human body and plays a central role in controlling metabolism, growth, maturation, fertility, stress responses, and fetal development [130,131]. Microplastics (MPs) and nanoplastics (NPs) may disrupt endocrine function both directly, through interactions with hormone-producing cells and tissues, and indirectly, by acting as carriers of endocrine-disrupting chemicals [32,132,133,134,135,136]. Of particular concern are chemicals associated with plastics or adsorbed onto their surfaces, including bisphenol A, phthalates, brominated flame retardants, pesticides, heavy metals, and persistent organic pollutants [132,134,136]. These compounds may mimic endogenous hormones, antagonize their receptors, interfere with hormone synthesis, and modify signaling pathways involved in endocrine homeostasis [132,137].

In the context of dietary exposure, endocrine effects may result from both the systemic bioavailability of ingested MNPs and the release or transport of plastic-associated chemicals within the gastrointestinal tract and after systemic distribution. Furthermore, MNP-induced alterations in the gut microbiota and intestinal barrier may indirectly influence endocrine regulation through interactions between microbial metabolites, inflammation, and metabolic signaling.

### 8.1. Disruption of Endocrine Function

Micro- and nanoplastics may affect endocrine regulation at multiple levels, involving both central regulatory structures and peripheral endocrine glands [32,132,135,138]. Particular attention has been directed toward the hypothalamic–pituitary–gonadal, hypothalamic–pituitary–thyroid, and hypothalamic–pituitary–adrenal axes [131,135,136,139]. Disruption of these regulatory systems may alter the secretion and activity of sex hormones, thyroid hormones, glucocorticoids, and hormones involved in growth and metabolic regulation [131,136,139].

At the hypothalamic and pituitary levels, MNP exposure may affect the expression of neurohormones and tropic hormones [131,139]. Experimental studies have reported reduced expression of gonadotropin-releasing hormone and alterations in luteinizing hormone (LH) and follicle-stimulating hormone (FSH) secretion, potentially resulting in secondary disturbances in gonadal function [131,136,140]. Neuroendocrine effects may also involve alterations in the secretion of oxytocin and vasopressin, which participate not only in reproductive regulation but also in social behavior, fluid homeostasis, and stress responses [135].

The adrenal glands may represent another target of MNP-associated endocrine toxicity [132,138,139]. Exposure to plastic particles and plastic-associated chemicals may disrupt cortisol, aldosterone, and other adrenal steroid secretion [135,139]. Such alterations may impair physiological stress responses, fluid and electrolyte balance, and the regulation of metabolic and immune processes [132,139]. Morphological alterations, including adrenal cortical hypertrophy, have also been described in experimental studies, potentially reflecting prolonged activation or dysfunction of the hypothalamic–pituitary–adrenal axis [135,139].

Experimental evidence suggests that MNPs and associated endocrine-disrupting chemicals may alter pancreatic and adipose tissue function, including pathways involved in adipocyte differentiation, lipid storage, and insulin signaling [132,136,139,141]. These findings indicate potential effects on metabolic regulation; however, evidence directly linking MNP exposure to insulin resistance, obesity, or type 2 diabetes in humans remains insufficient [132,135,136,141].

Pregnancy represents a particularly sensitive exposure window [32,135]. Micro- and nanoplastics may accumulate within the placenta, which acts as an essential endocrine organ regulating pregnancy maintenance and fetal development [13,132]. Placental dysfunction may interfere with the secretion of human chorionic gonadotropin, progesterone, estrogens, and placental lactogen [133,139]. Such disturbances may adversely affect implantation, fetal growth, and pregnancy outcomes [32].

### 8.2. Effects on Sex and Thyroid Hormones

Sex hormones and thyroid hormones represent major potential targets of MNP exposure and associated endocrine-disrupting chemicals [32,134,139]. In males, exposure to MNPs has been associated with reduced testosterone concentrations and altered LH and FSH secretion [132,140]. These effects may result from disruption of the hypothalamic–pituitary–testicular axis as well as direct effects on Leydig and Sertoli cells [131,142]. Reduced testosterone levels may impair spermatogenesis and contribute to lower sperm counts, reduced motility, and increased morphological abnormalities [130,132,138].

In females, MNPs may interfere with ovarian function, follicular development, steroidogenesis, and oocyte quality [132,135,136]. Experimental evidence suggests that exposure may promote follicular atresia and granulosa cell apoptosis and potentially reduce ovarian reserve. Disturbances in estradiol and progesterone synthesis may subsequently affect menstrual cyclicity, ovulation, and endometrial receptivity. These endocrine alterations may therefore contribute to impaired reproductive function, although the magnitude of these effects under typical human dietary exposure remains insufficiently established.

The thyroid gland represents another potentially sensitive target of endocrine disruption. Thyroid hormones, particularly triiodothyronine (T3) and thyroxine (T4), regulate energy metabolism, thermogenesis, neurological development, and cardiovascular function [132,135]. MNPs and associated chemicals may interfere with thyroid hormone synthesis, transport, metabolism, receptor signaling, and the enzymatic conversion of T4 to T3 [134,136,138].

Experimental studies have reported alterations in circulating thyroid-stimulating hormone (TSH), T3, and T4 concentrations, suggesting disruption of feedback regulation within the hypothalamic–pituitary–thyroid axis [132,139,141]. The gut microbiota may additionally contribute to these effects because MNP-induced dysbiosis can modify microbial metabolic activity and potentially influence thyroid hormone metabolism and endocrine signaling [13,132]. Such disturbances may be particularly important during prenatal development and childhood, when adequate thyroid hormone availability is critical for normal brain development [132,141].

### 8.3. Mechanisms of Endocrine Disruption

The endocrine effects associated with micro- and nanoplastics likely arise from the combined toxicity of the particles themselves and plastic-associated chemicals [132,134,139]. One of the most important mechanisms involves interactions with hormone receptors [136,137]. Compounds such as bisphenol A and phthalates may interact with estrogen, androgen, thyroid, and peroxisome proliferator-activated receptors (PPARs) [132,133,134]. Depending on the compound and biological context, they may act as agonists, antagonists, or receptor modulators, thereby altering hormone-dependent gene expression [32,135,137].

The previously described oxidative, mitochondrial, and inflammatory responses may also contribute to endocrine toxicity [32,132,136,138,143,144]. Of particular relevance, mitochondrial dysfunction in steroidogenic cells may impair steroid hormone biosynthesis, while inflammatory signaling may interfere with endocrine gland function and steroidogenesis [32,132,133,134,136].

Epigenetic modifications represent another potential mechanism [132,136]. Exposure to MNPs and plastic-associated chemicals may alter DNA methylation patterns, histone modifications, and microRNA expression [132,134]. Such changes may influence the expression of genes involved in development, sexual maturation, fertility, and metabolic regulation [134]. Some epigenetic alterations may persist over prolonged periods and potentially contribute to intergenerational or transgenerational effects [130,132].

The previously described carrier effect of MNPs may additionally contribute to endocrine disruption by facilitating tissue exposure to adsorbed endocrine-disrupting contaminants [132,134,136,139]. This mechanism may be particularly relevant in lipid-rich and metabolically active tissues, including the gonads, brain, thyroid gland, and placenta [130,132,134].

An additional challenge for toxicological risk assessment is the possibility of non-monotonic dose–response relationships, which are characteristic of many endocrine-disrupting chemicals [132,136]. Low exposure levels may produce biologically relevant effects that cannot necessarily be predicted from responses observed at higher doses [130,136]. Dose–response curves may therefore exhibit U-shaped or inverted U-shaped patterns, complicating the establishment of clear thresholds of biological safety [132,134]. Consequently, the endocrine effects of chronic MNP exposure require cautious interpretation and further investigation under exposure conditions relevant to the human diet [132,136,143].

To facilitate direct comparison of the experimental and human evidence discussed in Section 2, Section 3, Section 4, Section 5, Section 6, Section 7 and Section 8, representative studies are summarized in Table 4 according to polymer type, particle size, experimental model, principal biological effect, and observed disturbance.

## 9. Microplastics and Nanoplastics as Potential Carcinogenic Factors

Microplastics (MPs) and nanoplastics (NPs) are increasingly being investigated as potential contributors to carcinogenic processes [145,146,147,148]. Their potential significance is related to their widespread environmental occurrence, ability to cross biological barriers, and capacity for tissue accumulation [146,149,150,151]. Plastic particles have been detected in the lungs, blood, gastrointestinal tract, placenta, breast milk, and various tumor tissues [145,147,149,152]. Although current evidence is insufficient to classify MNPs as direct human carcinogens, a growing body of research suggests that they may contribute to tumor initiation, promotion, and progression through oxidative stress, chronic inflammation, endocrine disruption, DNA damage, and the transport of other toxic substances [145,150,153,154,155].

In the context of dietary exposure, the gastrointestinal tract may be particularly relevant because ingested particles are in prolonged contact with the intestinal epithelium and may alter barrier integrity and microbiota composition before a fraction of sufficiently small particles enters the systemic circulation. Consequently, potential carcinogenic effects may involve both local gastrointestinal mechanisms and systemic processes following particle translocation.

### 9.1. Mechanisms Potentially Involved in Carcinogenesis

The potential pro-carcinogenic effects of micro- and nanoplastics are likely to involve multiple interacting mechanisms. The previously described oxidative effects of MNPs may be particularly relevant to carcinogenesis through their capacity to induce DNA damage, including strand breaks and oxidative base modifications such as 8-oxoguanine [37,146,148,150,151,155,156]. If unrepaired, such lesions may become fixed as mutations during cell division, providing a biologically plausible mechanism through which MNP exposure could contribute to the initiation stage of carcinogenesis [145,147,157].

The previously described inflammatory effects of MNPs may also be relevant to carcinogenesis by promoting a persistent inflammatory microenvironment [145,146,148,154,155,157]. Such conditions may favor cellular proliferation, angiogenesis, extracellular matrix remodeling, and resistance to apoptosis, thereby providing a biologically plausible link to tumor promotion and progression [146,156,157].

MNPs may also interfere with intracellular signaling pathways regulating cell proliferation, survival, and migration [149,150,155]. Alterations involving NF-κB, MAPK/ERK, PI3K/Akt/mTOR, and Wnt/β-catenin signaling have been described [145,147,157]. These pathways are central to the regulation of inflammatory responses, metabolism, cell-cycle progression, and epithelial–mesenchymal transition [146,157]. Their persistent dysregulation could theoretically favor uncontrolled proliferation, increased invasiveness, and resistance to programmed cell death [147,156,157].

The previously described carrier function of MNPs may also be relevant to carcinogenesis by facilitating tissue exposure to adsorbed toxicants and potential carcinogens [37,145,146,147,149,150,154]. This mechanism could enhance local toxic and genotoxic effects; however, its contribution to cancer development under realistic human exposure conditions remains uncertain.

Endocrine disruption represents another mechanism of potential relevance [133,154]. Plastic-associated chemicals, including bisphenol A and phthalates, can interact with estrogen, androgen, and other nuclear receptors [37,145,147]. Such interactions may be particularly relevant to hormone-dependent cancers, including breast, prostate, ovarian, and endometrial cancers [145,155]. MNP exposure may also influence epigenetic regulation through changes in DNA methylation, histone modifications, and microRNA expression, potentially altering the expression of genes involved in proliferation, DNA repair, and apoptosis [145,147,150].

### 9.2. Experimental and Human Evidence

Current evidence regarding the carcinogenic potential of micro- and nanoplastics is derived predominantly from in vitro experiments and animal models [145,147]. Human epidemiological evidence remains limited, although emerging studies have reported associations between the presence of plastic particles in tissues and malignant disease [150,154,155]. MNPs have been detected in tumor tissues from the lungs, stomach, colon, prostate, breast, cervix, and other organs [145,155,157]. In some analyses, particle concentrations were higher in malignant tissues than in adjacent non-malignant tissues, suggesting that MNPs may be preferentially retained within the tumor microenvironment [145,147,158]. However, these findings cannot determine whether MNP accumulation precedes tumor development or occurs secondarily as a consequence of tumor-associated changes in tissue architecture, vascular permeability, or local clearance. Therefore, the higher particle burden observed in malignant tissues should be interpreted as an association rather than evidence of a carcinogenic effect. Some clinical observations have also suggested associations between greater tissue burdens of microplastics and more advanced disease characteristics, including lymph node involvement or poorer prognosis [145,158]. However, these findings are correlational and do not establish a causal relationship [145,150,154]. It remains possible both that MNPs contribute to tumor progression and that malignant tissues retain particles more readily because of altered vascularization, disrupted tissue architecture, and chronic inflammation [145,158].

Animal studies provide more direct mechanistic evidence [145,149,150]. In rodent models, exposure to polystyrene nanoplastics has been associated with enhanced tumor growth, alterations in the tumor microenvironment, activation of inflammatory pathways, and impaired antitumor immunity [146,149,157]. In aquatic models such as zebrafish, exposure to plastic particles has been linked to altered expression of genes involved in proliferation, apoptosis, oxidative stress, and carcinogenesis [150,155]. These findings indicate that MNPs may influence biological conditions that support tumor development and progression [145,147,159].

In vitro studies further demonstrate that both malignant and non-malignant cells can internalize micro- and nanoplastics [55,149,153,155]. Nanoplastics appear particularly capable of entering the cytoplasm and reaching lysosomes and mitochondria, while potential interactions with nuclear structures have also been proposed [55,145,149,160]. Exposure to polystyrene, polyethylene, and polypropylene particles has been associated with increased proliferation, migration, invasiveness, oxidative stress, DNA damage, and altered expression of genes involved in epithelial–mesenchymal transition [37,161,162]. Some studies have additionally suggested increased resistance to selected anticancer drugs, which could have implications for therapeutic responses [145,153,156].

### 9.3. Potential Contribution to Cancer Development and Progression

The potential contribution of micro- and nanoplastics to cancer should be considered as a chronic and multistage process [145,149,150,156]. These particles may interact with the human body over prolonged periods, while potential biological consequences may become evident only after sustained exposure [145,150]. During tumor initiation, MNPs may contribute to DNA damage and mutagenic processes [37,154,155]. During promotion, they may sustain inflammation and stimulate cellular proliferation [149,156]. During progression, they may potentially enhance angiogenesis, epithelial–mesenchymal transition, invasiveness, and metastatic capacity [145,157].

From the perspective of dietary exposure, the gastrointestinal tract may represent one of the most relevant sites of potential long-term carcinogenic interaction [149,158,161]. Ingested particles remain in direct contact with the intestinal mucosa and may impair the mucus layer, increase intestinal permeability, and promote gut microbiota dysbiosis [149,150]. Microbial alterations may favor pro-inflammatory and potentially genotoxic microbial profiles, thereby increasing susceptibility to chronic intestinal inflammation and possibly contributing to colorectal carcinogenesis [145,157]. For this reason, dietary MNP exposure has been proposed as one of several potential environmental factors that may warrant investigation in relation to the increasing incidence of early-onset colorectal cancer, although this hypothesis remains unproven [149,154,156].

Inhalation represents an additional relevant exposure route in relation to pulmonary carcinogenesis [151,157]. Airborne MNPs may deposit in the respiratory tract and alveoli, where they can promote chronic inflammation, oxidative stress, and impaired epithelial repair [37,145,146]. Their surfaces may also transport airborne contaminants with known carcinogenic potential, potentially increasing local toxicological burden [147,149,151].

For hormone-dependent cancers, plastic-associated endocrine-disrupting chemicals may be particularly relevant [155,156,163]. Bisphenol A, phthalates, and other additives can interfere with hormone receptors and alter the expression of genes regulating proliferation in hormonally responsive tissues [133,157]. Such mechanisms could theoretically contribute to the development or progression of breast, prostate, ovarian, and endometrial cancers, particularly during sensitive exposure windows such as fetal development, puberty, and pregnancy [150,155,164].

Overall, current evidence supports the biological plausibility of a potential role of MNPs in carcinogenic processes, but it remains insufficient to establish a causal relationship with cancer in humans. Most available evidence is derived from in vitro and animal studies, while human studies are predominantly observational or cross-sectional and therefore cannot establish temporality or exclude reverse causation and residual confounding. In addition, substantial heterogeneity in particle size, polymer type, analytical methods, exposure assessment, and experimental doses limits direct comparison between studies and complicates extrapolation to realistic human exposure. Establishing causality will require well-designed prospective longitudinal studies with standardized quantification of MNP exposure, repeated assessment of internal particle burden, adequate control of major confounders, evaluation of dose–response relationships, and long-term follow-up for incident cancer outcomes. Mechanistic studies using environmentally relevant exposure levels should complement such epidemiological evidence to determine whether MNP exposure precedes and contributes independently to carcinogenic processes. The current evidence supporting the potential involvement of MNPs in carcinogenic processes, together with the major limitations preventing causal inference, is summarized in Table 5.

**Figure 2:**
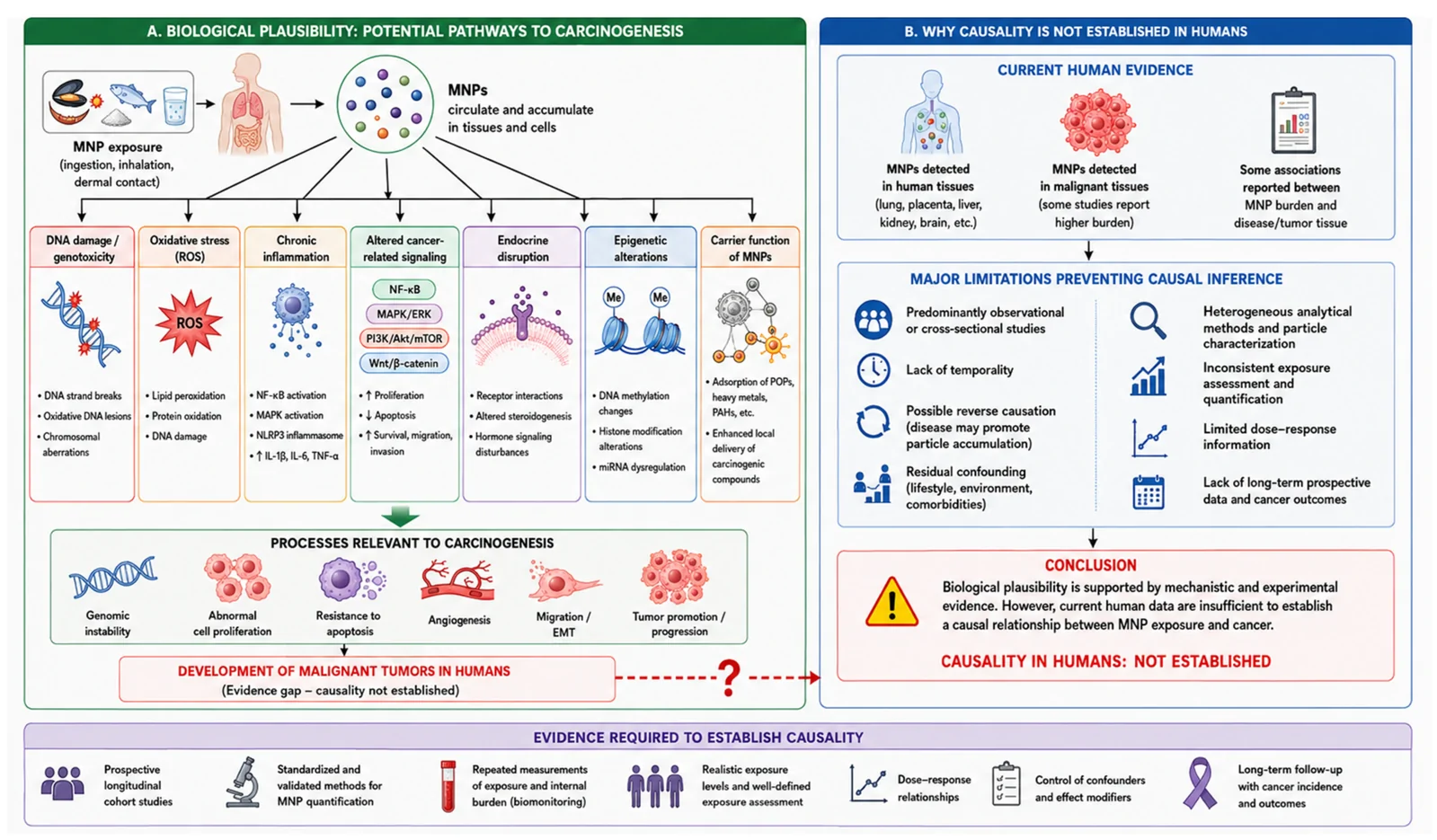
Proposed pathways linking microplastic and nanoplastic exposure to carcinogenesis and current limitations in establishing causality in humans. **Explanation:** ↑, increase; ↓, decrease; ?, uncertainty/evidence gap regarding causality in humans. Green indicates biological plausibility and potential pathways to carcinogenesis; blue indicates current human evidence and limitations to causal inference; red highlights the lack of established causality in humans; purple indicates evidence required to establish causality. Me, methyl group.

The proposed mechanisms linking MNP exposure with carcinogenic processes and the major evidence gaps preventing causal inference in humans are illustrated in Figure 2.

## 10. Effects of Microplastics and Nanoplastics on the Skeletal System

The skeletal system is increasingly recognized as a potential target of the adverse effects of microplastics (MPs) and nanoplastics (NPs) [165,166,167,168]. Following entry into the body, including through the gastrointestinal tract, smaller particles may cross biological barriers, enter the systemic circulation, and reach highly vascularized structures such as the bone marrow [165,169]. Plastic particles have also been detected in tissues of the musculoskeletal system, including cartilage and intervertebral disks, indicating that these structures may be susceptible to the systemic distribution and retention of MNPs [167,168,169]. Particular concern is associated with the potential long-term effects of MNPs on cells responsible for maintaining skeletal homeostasis, as disruption of the balance between bone formation and resorption may progressively impair bone structure and mechanical properties [165,168].

### 10.1. Effects on Bone Metabolism

Experimental evidence suggests that the previously described oxidative, inflammatory, and mitochondrial responses to MNP exposure may also disrupt skeletal homeostasis [16,166,168,169,170,171,172,173,174]. These alterations may impair the function of osteoblasts, chondrocytes, and bone marrow mesenchymal stem cells, potentially affecting extracellular matrix synthesis, mineralization, and tissue regeneration [16,165,166,168,169,175]. Collectively, such effects may disturb the physiological balance between bone formation and resorption; however, their relevance to skeletal health under typical human exposure conditions remains uncertain [167,168,169].

MNP exposure may also interfere with the differentiation of bone marrow mesenchymal stem cells [165,166]. Experimental evidence suggests that exposure may shift their differentiation toward adipogenesis at the expense of osteogenesis, thereby reducing the pool of cells capable of developing into bone-forming osteoblasts [166,168]. Such alterations could impair bone regenerative capacity and contribute to deterioration of bone quality during prolonged exposure [165,168,172].

The gut–bone axis may represent an additional pathway linking dietary exposure to MNPs with skeletal effects [165,169]. MNP-induced alterations in the gut microbiota and intestinal barrier may promote systemic inflammation and potentially affect mechanisms involved in calcium metabolism, vitamin D homeostasis, and immune regulation of bone remodeling [165,169,171]. Plastic-associated chemicals, including bisphenols and phthalates, may additionally interfere with hormonal pathways involved in bone growth, remodeling, and mineralization [16,165,176].

### 10.2. Alterations in Bone Remodeling and Mineralization

Physiological bone remodeling depends on a tightly regulated balance between osteoblast-mediated bone formation and osteoclast-mediated bone resorption [165,175,176]. Experimental evidence indicates that MNPs may disrupt this balance by suppressing osteoblast function while promoting osteoclast activity [165,168]. Persistent disruption of these processes could favor excessive bone resorption and contribute to deterioration of bone microarchitecture and mineralization [165,172,174].

Enhanced bone resorption may involve alterations in the receptor activator of nuclear factor-κB ligand/osteoprotegerin (RANKL/OPG) signaling axis, a major regulator of osteoclast differentiation and activity [165,168,175]. Increased RANKL-mediated signaling may promote osteoclastogenesis and enhance the expression of markers associated with bone matrix degradation [165,175]. In parallel, MNPs may inhibit osteogenesis by interfering with signaling pathways involved in osteoblast differentiation, including BMP/Smad and Wnt/β-catenin pathways [168,175,177]. Altered expression of osteogenic regulators, including Runx2 and Osterix, together with reduced alkaline phosphatase activity, may consequently impair extracellular matrix mineralization and new bone formation [128,165,169,177].

Collectively, these experimental findings raise concerns about potential skeletal effects of chronic MNP exposure; however, direct evidence linking environmental or dietary exposure to osteoporosis, reduced bone mineral density, fracture risk, or degenerative joint disease in humans remains insufficient [165,168,176]. Experimental studies have additionally reported alterations in cartilage homeostasis, intervertebral disk integrity, and skeletal development [166,167,169,170,172,173,174,177]. Whether these effects occur in humans at exposure levels relevant to the general population remains to be established.

## 11. Discussion

The available evidence indicates that microplastics (MPs) and nanoplastics (NPs) represent emerging contaminants of potential relevance to human health. Dietary exposure through contaminated food and drinking water is particularly important because ingested particles may interact with the gastrointestinal tract and, especially in the case of smaller particles, cross biological barriers and enter the systemic circulation. Their detection in blood and multiple human tissues supports the possibility of systemic distribution, tissue accumulation, and prolonged biological interactions.

**Figure 3:**
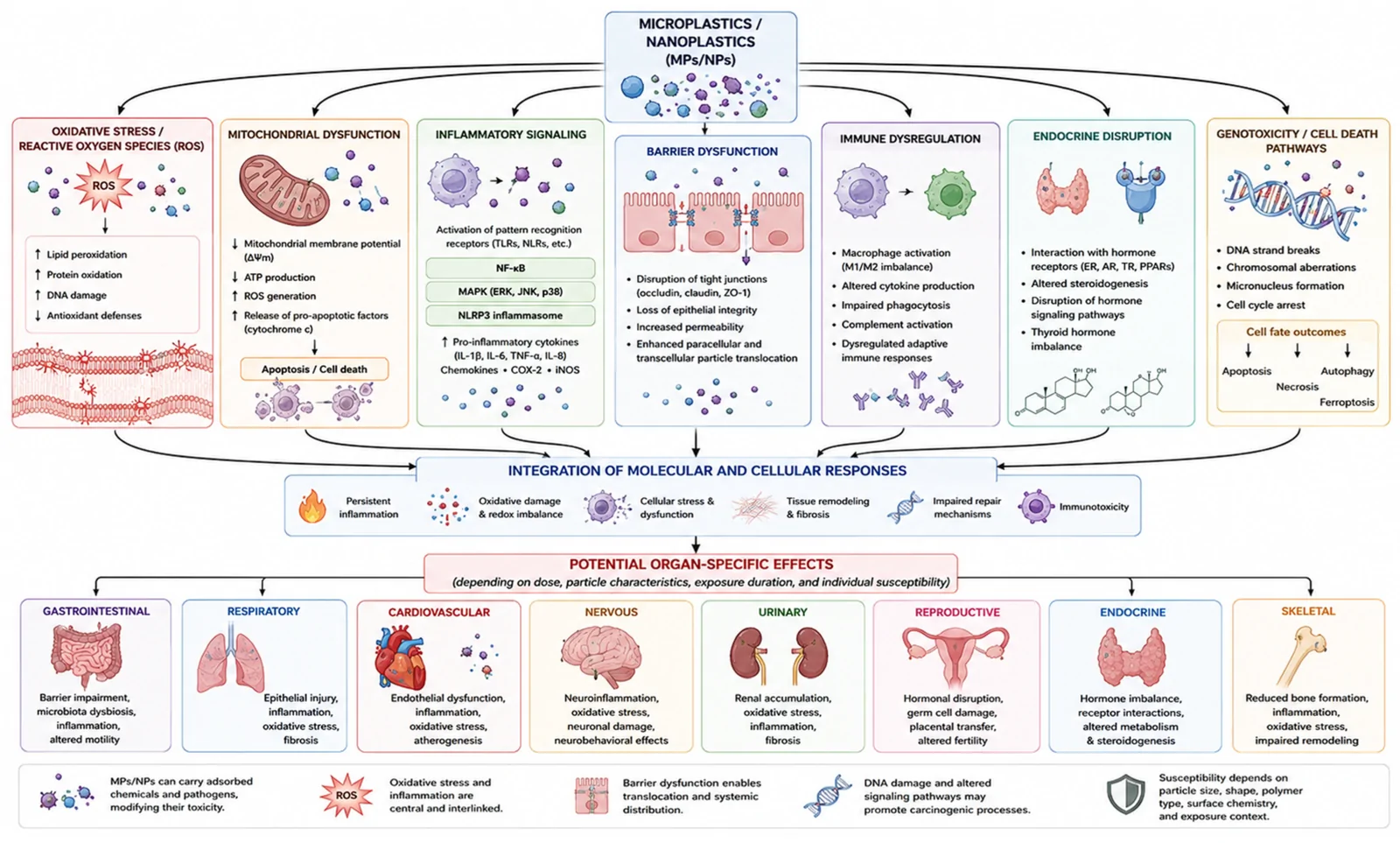
Interconnected molecular and cellular mechanisms potentially involved in microplastic- and nanoplastic-induced toxicity. **Explanation:** ↑, increase; ↓, decrease. Different colors distinguish the major molecular and cellular pathways and organ-specific effects presented in the figure and are used solely to facilitate visual differentiation; they do not represent different levels of evidence or effect magnitude.

Across the organ systems evaluated in this review, experimental studies consistently identified several interconnected mechanisms, particularly oxidative stress, inflammation, mitochondrial dysfunction, barrier impairment, genotoxicity, immune dysregulation, and endocrine disruption. Rather than representing organ-specific pathways, these mechanisms appear to constitute recurring biological responses to MNP exposure, while their magnitude and clinical relevance in humans remain incompletely established. The gastrointestinal tract may be particularly relevant to dietary exposure by mediating particle uptake and systemic effects associated with intestinal barrier impairment and gut microbiota dysbiosis. The principal interconnected molecular and cellular mechanisms potentially involved in MNP-induced toxicity and their relationship with organ-specific effects are summarized in Figure 3.

However, interpretation of the available evidence remains challenging. The biological behavior and toxicity of MNPs depend strongly on particle size, shape, polymer type, surface properties, and associated chemical contaminants. More specifically, particle size is a major determinant of cellular uptake and translocation. Smaller particles generally show greater cellular uptake and a higher capacity to cross biological barriers; for example, uptake of PS particles by Caco-2 cells decreased from 78% for 300 nm particles to 28% for 6 μm particles. Particles below approximately 10–20 μm have been reported to cross biological membranes or access human tissues, while nanoscale particles show particularly high translocation potential. Polymer composition may also influence biological interactions; commonly investigated polymers include PS, PE, PP, PET, and PVC, although direct comparisons between polymer types remain limited and PS is disproportionately represented in experimental studies. Surface properties further modify biological responses, as surface charge, functionalization, hydrophobicity, and biomolecular corona formation can affect membrane interactions, cellular uptake, and toxicity. In particular, amino-functionalized PS particles have been associated with membrane destabilization, ROS generation, lysosomal damage, and increased cytotoxicity, while surface functionalization can substantially alter cellular internalization. Collectively, these findings indicate that MNP toxicity cannot be attributed to particle size or polymer identity alone but rather results from interactions among multiple physicochemical characteristics and exposure conditions.

Importantly, many experimental studies use standardized particles, relatively high exposure concentrations, and exposure conditions that may not adequately reflect the heterogeneous and generally lower-level exposures experienced by humans. This limits quantitative extrapolation of experimental findings to human health risk. In addition, the presence of plastic additives and adsorbed environmental contaminants complicates attribution of observed effects specifically to the particles themselves.

Current evidence suggests that particle size is one of the most important determinants of the biological activity of MNPs. In general, NPs appear to have a greater toxicological potential than larger MPs because their smaller dimensions and higher surface-area-to-volume ratio facilitate cellular uptake, translocation across biological barriers, interaction with intracellular structures, and systemic distribution. Particles in the nanoscale range, particularly those below approximately 100 nm, may therefore represent a fraction of particular concern. However, it is currently not possible to define a specific particle size or polymer as the most toxic to the human body. Toxicity depends not only on size but also on polymer composition, shape, surface charge and functionalization, environmental aging, adsorbed contaminants, and chemical additives. Although experimental studies frequently report adverse effects for PS, PE, PET, and PVC particles, direct comparisons between polymers under standardized exposure conditions remain scarce. Consequently, the available evidence supports a higher biological hazard potential of smaller particles, particularly NPs, but does not currently allow a robust ranking of individual MNP types according to their toxicity in humans.

Although associations between MNP accumulation and several pathological conditions have been reported, current human evidence remains insufficient to establish direct causal relationships. Most mechanistic knowledge is derived from in vitro and animal studies, while long-term epidemiological data remain limited. Considerable methodological differences in particle detection, identification, and quantification also restrict comparisons between studies, particularly in the case of nanoplastics.

Future research should therefore focus on standardized analytical methods, realistic chronic exposure models, improved detection of nanoplastics, and prospective human studies capable of linking exposure levels with clinically relevant health outcomes. Particular attention should also be given to long-term low-dose exposure and vulnerable populations. Such studies are necessary to determine the actual contribution of MNP exposure to human disease and to support reliable health risk assessment.

From a public-health perspective, improved analytical detection of NPs should be regarded primarily as a tool for more accurate exposure assessment rather than as a strategy for reducing exposure itself. Effective mitigation should focus on limiting particle release at the source, particularly from food-contact materials. Where technologically and environmentally appropriate, reducing direct food contact with conventional plastics and increasing the use of alternative materials, including glass, stainless steel, paper-based systems, or appropriately designed bio-based materials, may contribute to lower MNP release. Glass may be particularly relevant for selected beverages and foods because it does not generate plastic particles during normal use; however, no packaging material should currently be considered universally superior, as food safety, chemical migration, product stability, energy requirements, transportation, recyclability, and life-cycle environmental impacts must also be considered. Moreover, biodegradable or bio-based polymers should not automatically be assumed to eliminate MNP exposure because they may also undergo fragmentation. Therefore, future strategies should combine improved MNP detection with the development and validation of low-migration food-contact materials, optimization of packaging design and processing conditions, and comparative studies evaluating particle release from conventional plastics and alternative packaging under realistic conditions of use.

## 12. Materials and Methods

The present narrative review was prepared based on scientific literature identified through a structured search of the PubMed/MEDLINE, Scopus, Web of Science, and Google Scholar databases. Publications published between 2000 and 2026 were considered, with particular emphasis on recent studies addressing human exposure to microplastics (MPs) and nanoplastics (NPs), dietary sources, gastrointestinal bioavailability, toxicokinetics, and potential systemic health effects. The literature search was conducted using Boolean operators (“AND”, “OR”) in combination with the following keywords: microplastics, nanoplastics, dietary exposure, food contamination, food contact materials, drinking water, gastrointestinal absorption, bioavailability, translocation, toxicokinetics, biodistribution, bioaccumulation, elimination, human tissues, gut microbiota, intestinal barrier, oxidative stress, inflammation, mitochondrial dysfunction, genotoxicity, endocrine disruption, organ toxicity, carcinogenicity, and human health. Reference lists of eligible publications were additionally screened to identify further relevant studies.

The inclusion criteria comprised original research articles and review papers that provided relevant information on: (i) dietary exposure to microplastics (MPs) and nanoplastics (NPs); (ii) their occurrence in food and drinking water; (iii) migration from food-contact materials; (iv) gastrointestinal absorption, bioavailability, translocation, biodistribution, bioaccumulation, and elimination; (v) molecular mechanisms of toxicity; and (vi) potential organ-specific and systemic health effects. Full-text publications written in English or Polish were considered eligible. Studies addressing non-dietary exposure routes were also included when they provided relevant comparative information concerning total human exposure, systemic distribution, or toxicity.

The exclusion criteria comprised conference abstracts without full-text availability, conference proceedings, editorials, commentaries, duplicate publications, studies lacking sufficient scientific relevance to the objectives of the review, and publications that did not directly address human exposure, toxicokinetics, biological effects, or health-related consequences of MPs and NPs. Studies focused exclusively on environmental occurrence or ecotoxicological effects without relevance to human exposure or health were also excluded.

The selected publications were subjected to qualitative analysis according to the predefined thematic scope of the review. Information was extracted on major sources of exposure, gastrointestinal uptake, translocation across biological barriers, systemic distribution, tissue accumulation, and elimination of MNPs. Evidence concerning the molecular mechanisms of toxicity, including oxidative stress, inflammation, mitochondrial dysfunction, genotoxicity, and endocrine disruption, was also evaluated. Particular attention was given to potential effects on the gastrointestinal, respiratory, cardiovascular, nervous, urinary, reproductive, endocrine, and skeletal systems, as well as the possible involvement of MNPs in carcinogenic processes.

As this study was conducted as a narrative literature review and did not involve human participants, identifiable personal data, or experimental procedures, approval from an ethics committee and study registration were not required.

## 13. Conclusions

Microplastics and nanoplastics represent widespread contaminants to which humans are continuously exposed, with food and drinking water constituting important sources of exposure. Available evidence indicates that sufficiently small particles may cross biological barriers, enter the systemic circulation, and reach multiple organs and tissues. Their potential toxicity involves interconnected mechanisms, particularly oxidative stress, chronic inflammation, mitochondrial dysfunction, disruption of biological barriers, genotoxicity, immune dysregulation, and endocrine disruption.

Current evidence indicates that MNP exposure can induce biological responses relevant to multiple organ systems in experimental models; however, the extent to which these findings translate into clinically significant effects in humans remains uncertain. Most mechanistic evidence is derived from in vitro cellular models and in vivo animal studies, predominantly involving rodents, with additional evidence from models such as zebrafish, whereas human data remain limited and predominantly observational. Accordingly, the detection of MNPs in human tissues and reported associations with pathological conditions should not be interpreted as evidence that MNP exposure causes or contributes to the development or progression of human disease.

The major priorities for future research include the standardization of analytical methods, improved detection of nanoplastics, accurate assessment of real-world dietary exposure, and long-term prospective studies in humans. Particular attention should be given to chronic low-dose exposure, vulnerable population groups, and the combined effects of plastic particles, chemical additives, and adsorbed environmental contaminants. A more precise understanding of MNP bioavailability and toxicokinetics will be essential for establishing reliable health-based guidance values and developing effective strategies to reduce human exposure.

## Figures and Tables

**Table 1:** Major dietary sources and pathways of human exposure to microplastics and nanoplastics.

Exposure Source	Examples	Main Source/Pathway of Contamination	Relevance to Human Exposure	References
**Fish and seafood**	Fish, shellfish and other seafood products	Environmental contamination of aquatic ecosystems and subsequent presence of MNPs in edible organisms	Represents a direct dietary pathway of MNP exposure through consumption of contaminated aquatic foods	[2,3,21,28,34,35,36,37,38,39,40]
**Drinking water**	Tap/municipal water, bottled water	Environmental contamination; additional contribution may arise from plastic packaging	Frequently reported dietary source; exposure varies with water source and quality	[2,3,21,28,34,35,36,37,38,39,40]
**Table salt**	Commercial table salt	Environmental contamination during production and processing	Provides a recurring dietary source because of regular consumption	[2,3,21,28]
**Milk and other beverages**	Milk and beverages	Environmental contamination, processing, storage, and contact with plastic materials	Contributes to repeated oral exposure to MNPs	[2,3,21,28]
**Honey**	Honey	Environmental contamination occurring along the production chain	Additional food-related source of oral MNP exposure	[2,3,21,28]
**Food-contact materials**	Plastic packaging, containers and other materials contacting food	Release and migration of plastic particles during storage and thermal processing	May increase contamination of otherwise consumed food and beverages	[4,10,29]
**Contamination during the food chain**	Food contaminated during production, transportation or storage	Secondary contamination associated with processing, handling, transport and storage	Broad indirect pathway contributing to dietary exposure across different food categories	[34,35,37,38,39,40]

**Table 2:** Toxicokinetic behavior of microplastics and nanoplastics following human exposure.

Toxicokinetic Process	Main Observations	Factors/Mechanisms Involved	Potential Biological Relevance	References
**Gastrointestinal absorption**	Most larger particles remain within the gastrointestinal tract, whereas smaller MPs and NPs show greater potential for intestinal uptake	Particle size, shape, chemical composition and surface properties; uptake through M cells and enterocytes	Determines the fraction potentially available for systemic translocation	[3,5,10,18,19,21,25]
**Systemic translocation**	Smaller particles, particularly NPs, may cross biological barriers and enter blood and lymphatic circulation	Transcellular/endocytic transport and passage across biological barriers	Enables transport from the primary site of exposure to distant tissues	[3,5,18,32]
**Biodistribution**	MNPs have been reported in human blood and multiple tissues, including lungs, liver, spleen, kidneys, heart, placenta and brain	Transport through blood and lymphatic systems	Indicates the possibility of systemic distribution following exposure	[3,4,14,18,20,32]
**Bioaccumulation and persistence**	MNPs may persist within biological tissues	Chemical stability and resistance to enzymatic degradation	May prolong particle–tissue interactions	[3,21,23]
**Transplacental and neurological translocation**	Experimental evidence indicates that NPs may cross placental and blood–brain barriers	Small particle size and ability to cross specialized biological barriers	Raises concern regarding exposure of protected tissues and developmental stages	[4,14,18,20]
**Gastrointestinal elimination**	The majority of ingested particles are eliminated in feces	Limited absorption of larger particles and gastrointestinal transit	Represents the principal elimination pathway after oral exposure	[2,28,33]
**Urinary elimination**	Smaller particles may potentially undergo renal elimination	Filtration and urinary excretion dependent on particle characteristics	Possible secondary elimination pathway for sufficiently small particles	[5,21,23,27]
**Respiratory clearance**	Deposited respiratory particles may be removed from the airways	Mucociliary clearance and phagocytic activity of alveolar macrophages	Limits retention of a fraction of inhaled particles	[5,21,23,27]

**Table 3:** Summary of potential organ-specific effects of microplastic and nanoplastic exposure and the current strength of evidence.

Organ System	Main Reported Effects	Particularly Relevant Mechanisms	Predominant Evidence	Human Evidence	References
Gastrointestinal	Intestinal barrier impairment; gut microbiota dysbiosis; altered digestive function; local inflammatory responses	Barrier disruption; microbiota alterations; oxidative and inflammatory responses	In vitro; animal	MNP detection in gastrointestinal tissues; clinical causality not established	[34,35,36,37,38,39,40,41,42,43,44,45,46,47,48,49,50,51,52,53,54,55,56]
Respiratory	Epithelial injury; impaired pulmonary function; inflammatory and fibrotic responses; altered local immune defense	Epithelial barrier impairment; inflammation; oxidative injury; macrophage dysfunction	In vitro; animal	MNPs detected in human lung tissue; long-term clinical effects remain uncertain	[57,58,59,60,61,62,63,64,65,66,67,68,69,70,71]
Cardiovascular	Endothelial dysfunction; vascular inflammation; myocardial injury; potential contribution to atherosclerotic processes	Endothelial injury; oxidative/inflammatory responses; systemic particle distribution	In vitro; animal; observational human	Emerging observational evidence; causality not established	[72,73,74,75,76,77,78,79,80,81,82,83,84,85]
Nervous	Neuroinflammatory responses; neuronal dysfunction; potential impairment of neuroplasticity and neural repair	Blood–brain barrier translocation; neuroinflammation; mitochondrial dysfunction	Predominantly animal/in vitro	MNPs detected in human brain tissue; associations with neurological disease remain non-causal	[4,86,87,88,89,90,91,92,93,94,95,96,97,98,99,100]
Urinary	Renal accumulation; tubular and glomerular injury; altered renal function; potential fibrotic changes	Renal particle retention; oxidative/inflammatory responses; ER stress; ferroptosis	Predominantly animal/in vitro	MNPs detected in urine and renal tissue; clinical significance uncertain	[38,40,101,102,103,104,105,106,107,108,109,110,111]
Reproductive	Altered spermatogenesis and sperm quality; ovarian dysfunction; follicular alterations; potential developmental effects	Gonadal accumulation; endocrine disruption; germ-cell injury; placental translocation	Predominantly animal/in vitro	MNPs detected in reproductive tissues; direct effects on human fertility not established	[112,113,114,115,116,117,118,119,120,121,122,123,124,125,126,127,128,129]
Endocrine	Altered sex and thyroid hormone regulation; disruption of steroidogenesis and metabolic signaling	Hormone receptor interactions; endocrine-disrupting chemicals; altered steroidogenesis	Predominantly animal/in vitro	Human evidence limited; clinical endocrine consequences remain uncertain	[32,130,131,132,133,134,135,136,137,138,139,140,141,142,143,144,145,146,147,148,149,150,151,152,153,154,155,156,157,158,159,160,161,162,163,164]
Skeletal	Altered osteoblast/osteoclast activity; impaired mineralization; changes in cartilage and skeletal development	RANKL/OPG imbalance; altered osteogenesis; Wnt/β-catenin and BMP/Smad signaling	Predominantly animal/in vitro	Direct evidence for osteoporosis, fracture risk or degenerative disease in humans insufficient	[16,165,166,167,168,169,170,171,172,173,174,175,176,177]

**Table 4:** Comparative summary of representative studies investigating organ-specific effects of microplastics and nanoplastics.

Organ System	Polymer/Type	Particle Size	Research Model	Main Effect	Observed Disturbance	Ref.
Gastrointestinal	PET MPs, irregular particles	160 ± 110 µm	Dynamic simulated human gastrointestinal/colonic model with human microbiota	Gut microbiota disturbance	Altered colonic microbial community; microbial adhesion and biofilm formation on PET; changes in particle surface during digestion	[44]
Gastrointestinal	PS MPs	1 µm	Mice, oral exposure	Gut–liver axis dysfunction	Altered intestinal microbiota and metabolism, intestinal inflammation, increased fasting glucose and insulin, insulin resistance	[49]
Gastrointestinal	PS NPs	20 nm	BALB/c mice, AOM/DSS CAC model + Caco-2 cells	Oxidative/genotoxic and metabolic effects	↑ ROS, lipid peroxidation and DNA damage; impaired lipid metabolism; PI3K/AKT/mTOR involvement; increased tumor burden	[52]
Gastrointestinal/liver	PET, PVC, PP, PS and other MPs	4–30 µm	Human liver tissue	Tissue accumulation	Six polymer types detected; greater MP burden in cirrhotic liver than in controls	[46]
Respiratory	PET NPs	~136 nm	Human A549 lung epithelial cells	Oxidative stress and genotoxicity	~30% increase in ROS and increased DNA strand damage after exposure	[62]
Respiratory	MPs/NPs	MPs ~2.6 µm; NPs ~213 nm	C57BL/6 mice + BEAS-2B cells	Aggravation of asthmatic airway remodeling	Airway hyperresponsiveness, goblet-cell hyperplasia, ↑ IL-4/IL-5/IL-13; HSP90α-mediated PI3K–Akt–mTOR activation	[63]
Respiratory	PVC, PS, PET, PP, PMMA, PE, nylon MPs	~1–2.5 µm to >20 µm	Human BALF and lung tissue	Pulmonary accumulation/inflammatory association	MPs detected in BALF; particle burden positively associated with serum CRP	[65]
Cardiovascular	PE and PVC MNPs	predominantly <200 nm	304 carotid endarterectomy patients	Atherosclerotic accumulation	MNP-positive plaques associated with substantially higher risk of myocardial infarction, stroke or death	[81]
Cardiovascular	PVC, PS, PE, PA66 MNPs	NR	110 myocardial infarction patients	Cardiovascular event association	Higher PVC burden associated with inflammatory cytokines and major adverse cardiovascular events	[82]
Cardiovascular	PS MPs	NR	Rats	Cardiovascular toxicity	Metabolomic disturbances associated with cardiovascular injury	[83]
Nervous	PS MPs, spherical	2 µm	Male C57BL/6 mice	Neurotoxicity	Impaired hippocampus-dependent learning and memory, neuroinflammation, BBB alterations and reduction in synaptic proteins	[95]
Nervous	PS NPs	50 nm	C57BL/6J mice	Neurobehavioral toxicity	Aggravated seizure phenotype and impaired memory; hippocampal neuronal ferroptosis associated with iron/lipid metabolic disturbance	[99]
Urinary	PA, PE, PP, POM, PB1 MNPs	0.7–20 µm	50 healthy adults; urine samples	Association with renal-function biomarkers	Urinary MNP burden associated with increased urinary albumin-to-creatinine ratio	[104]
Reproductive	PS MPs/NPs, spherical	80 nm, 0.5 µm, 5 µm	Male mice	Male reproductive toxicity	Impaired spermatogenesis, oxidative stress, testicular inflammation, ↓ testosterone; size-dependent metabolic effects	[127]
Reproductive	PE, PVC, nylon-66, PP and other polymers	NR	Human and canine testes	Reproductive tissue accumulation	Multiple polymers detected in testes; polymer burden associated with testicular parameters in dogs	[120]
Reproductive/placental	PS NPs	20 and 100 nm	Primary human placental cytotrophoblasts	Cellular stress and placental toxicity	Cytotoxic/inflammatory effects and altered autophagic flux, including increased LC3-II/LC3-I and Beclin expression	[145]
Endocrine	PS MPs	NR	Male BALB/c mice	Thyroid dysfunction	Oxidative stress and thyroid histopathology; altered TSH/TPO/TSHR-related regulation and reduced circulating thyroid-axis markers	[142]
Endocrine/reproductive	PS MPs	NR	Sheep	HPG-axis disruption	↓ hypothalamic GnRH, KISS-1 and NKB; ↓ circulating LH and FSH	[131]
Endocrine	PVC, PP, PBS MPs	NR	Pregnant women/umbilical cord blood	Fetal endocrine association	Exposure associated with altered androgenic/glucocorticoid hormones, including lower cortisol/cortisone	[134]
Endocrine	Placental MPs	NR	Mother–newborn pairs	Neonatal thyroid association	Greater placental MP exposure associated with altered neonatal thyroid function, including lower T4	[129]

↑, increase; ↓, decrease.

**Table 5:** Current evidence concerning the potential involvement of microplastics and nanoplastics in carcinogenic processes and major limitations for causal inference.

Evidence Category	Main Findings	Relevance to Carcinogenesis	Major Limitations	References
Genotoxicity and DNA damage	DNA strand breaks, oxidative DNA lesions, and other genotoxic alterations have been reported following MNP exposure	Provides biological plausibility for processes relevant to cancer initiation	Evidence is predominantly experimental; exposure levels and particle characteristics may not reflect typical human exposure	[37,145,146,147,148,149,150,151,155,156,157]
Chronic inflammatory responses	MNP exposure may promote a persistent inflammatory microenvironment	May favor cellular proliferation, angiogenesis, extracellular matrix remodeling, and resistance to apoptosis	Predominantly mechanistic and experimental evidence; direct contribution to human tumor development remains unproven	[145,146,148,154,155,156,157]
Altered cancer-related signaling	Alterations involving NF-κB, MAPK/ERK, PI3K/Akt/mTOR, and Wnt/β-catenin pathways have been reported	These pathways regulate proliferation, survival, migration, and other processes relevant to tumor development and progression	Findings are largely derived from experimental models and cannot establish cancer risk in humans	[145,146,147,148,149,150,151,152,153,154,155,156,157]
Carrier function of MNPs	MNPs may transport adsorbed toxicants and potential carcinogens and modify their local bioavailability	Could enhance local toxic and genotoxic effects	Contribution under realistic human exposure conditions remains uncertain; effects of particles and associated chemicals are difficult to distinguish	[37,145,146,147,148,149,150,154]
Endocrine and epigenetic alterations	MNP-associated exposure may interfere with endocrine signaling and epigenetic regulation	Potentially relevant to hormone-dependent and other carcinogenic processes	Long-term significance and dose–response relationships in humans remain insufficiently characterized	[145,146,147,148,149,150,151,152,153,154,155,156,157]
MNP detection in human tumors	Plastic particles have been detected in several malignant human tissues, with some studies reporting higher particle burdens in tumors than in adjacent non-malignant tissues	Demonstrates particle presence and suggests possible preferential retention within the tumor microenvironment	Does not establish temporality or causality; tumor-associated changes may themselves promote particle accumulation, creating the possibility of reverse causation	[145,147,158]
Overall human evidence	Associations between MNP burden and malignant or pathological tissues have been reported	Supports further investigation of the potential relevance of MNP exposure to human carcinogenesis	Human evidence is mainly observational or cross-sectional; residual confounding, reverse causation, heterogeneous exposure assessment, and limited longitudinal data prevent causal inference	[145,146,147,148,149,150,151,152,153,154,155,156,157,158,159,160,161,162,163,164]

## Data Availability

No new data were created or analyzed in this study. Data sharing is not applicable to this article.
